# Synergistic Effects of Genetic Variants of Glucose Homeostasis and Lifelong Exposures to Cigarette Smoking, Female Hormones, and Dietary Fat Intake on Primary Colorectal Cancer Development in African and Hispanic/Latino American Women

**DOI:** 10.3389/fonc.2021.760243

**Published:** 2021-10-07

**Authors:** Su Yon Jung, Eric M. Sobel, Matteo Pellegrini, Herbert Yu, Jeanette C. Papp

**Affiliations:** ^1^ Translational Sciences Section, Jonsson Comprehensive Cancer Center, School of Nursing, University of California, Los Angeles, Los Angeles, CA, United States; ^2^ Department of Human Genetics, David Geffen School of Medicine, University of California, Los Angeles, Los Angeles, CA, United States; ^3^ Department of Computational Medicine, David Geffen School of Medicine, University of California, Los Angeles, Los Angeles, CA, United States; ^4^ Department of Molecular, Cell and Developmental Biology, Life Sciences Division, University of California, Los Angeles, Los Angeles, CA, United States; ^5^ Cancer Epidemiology Program, University of Hawaii Cancer Center, Honolulu, HI, United States

**Keywords:** glucose homeostasis, random survival forest, attributable risk, smoking, endogenous estrogen, polyunsaturated fatty acid, colorectal cancer, African and Hispanic/Latino American women

## Abstract

**Background:**

Disparities in cancer genomic science exist among racial/ethnic minorities. Particularly, African American (AA) and Hispanic/Latino American (HA) women, the 2 largest minorities, are underrepresented in genetic/genome-wide studies for cancers and their risk factors. We conducted on AA and HA postmenopausal women a genomic study for insulin resistance (IR), the main biologic mechanism underlying colorectal cancer (CRC) carcinogenesis owing to obesity.

**Methods:**

With 780 genome-wide IR-specific single-nucleotide polymorphisms (SNPs) among 4,692 AA and 1,986 HA women, we constructed a CRC-risk prediction model. Along with these SNPs, we incorporated CRC-associated lifestyles in the model of each group and detected the topmost influential genetic and lifestyle factors. Further, we estimated the attributable risk of the topmost risk factors shared by the groups to explore potential factors that differentiate CRC risk between these groups.

**Results:**

In both groups, we detected IR-SNPs in *PCSK1* (in AA) and *IFT172*, *GCKR*, and *NRBP1* (in HA) and risk lifestyles, including long lifetime exposures to cigarette smoking and endogenous female hormones and daily intake of polyunsaturated fatty acids (PFA), as the topmost predictive variables for CRC risk. Combinations of those top genetic- and lifestyle-markers synergistically increased CRC risk. Of those risk factors, dietary PFA intake and long lifetime exposure to female hormones may play a key role in mediating racial disparity of CRC incidence between AA and HA women.

**Conclusions:**

Our results may improve CRC risk prediction performance in those medically/scientifically underrepresented groups and lead to the development of genetically informed interventions for cancer prevention and therapeutic effort, thus contributing to reduced cancer disparities in those minority subpopulations.

## Introduction

Although cancer mortality has declined throughout all racial/ethnic groups since 1971 when the National Cancer Act, known as the “War on Cancer”, began, cancer health disparities still exist in the form of higher cancer incidence and mortality among the racial/ethnic minorities (1). In particular, colorectal cancer (CRC) incidence and death rates in African American (AA) women are highest among all racial/ethnic female groups and, compared with white women, 20% and 35%, respectively, were higher during 2012–2016 (2, 3). Also, in the 2 largest minorities, AA and Hispanic/Latino American (HA) women, CRC is the third leading cause of cancer diagnosis and related death (3, 4).

The risk for CRC development increases in older women. For example, approximately 90% of new CRC cases occur in women 50 years old and older (2), and one of the main risk factors is excessive adiposity (5, 6). Specifically, among AA and HA postmenopausal women of at least age 50 years, our preliminary analysis (**Table S1**) of abdominal adiposity (measured by waist circumference and waist-to-hip ratio) supported the role of obesity in increased risk for CRC, despite insufficient statistical power. For the major biologic mechanism of colorectal tumorigenesis due to obesity, insulin resistance (IR) or glucose intolerance has been thought to play a key mediating role (7, 8). Specifically, increased levels of glucose and insulin, reflecting IR, which interacts with obesity, promoted colorectal epithelial proliferation (9); the elevated insulin levels stimulated the growth of CRC in both cell lines (10) and an animal model (11). IR promotes mitosis by overexpressing insulin receptors and insulin-like growth factor 1 receptors and by dysregulating downstream cellular signaling cascades, resulting in enhancement of cellular anabolic status and increased anti-apoptosis and cell proliferation (12, 13). IR may thus initiate and facilitate CRC cell growth. However, studies focusing on AA and HA women for IR in relation to CRC risk are lacking. One study of DNA methylation in association with CRC among AAs (mainly women) (14) revealed aberrant methylation of CpG islands in the genes that are involved in an insulin network, suggesting the critical role of IR in AA women’s colorectal carcinogenesis. Also, the preliminary results (**Table S1**) in AA and HA women from our analysis of the fasting glucose and insulin levels (FG and FI) indicated that increased levels of both molecules (particularly glucose) were associated with higher risk for CRC in both groups, but these findings lacked sufficient power to reach significance.

Considering that the systemic development of IR can be influenced by not only environmental (15–17) but also genetic factors (18, 19), studying genomic markers that explain variations of glucose and insulin concentrations may provide more confirmatory understanding of those concentrations’ role in CRC development. The effort to detect genetic variations of IR has been made in extensive genomic studies, but they mostly focused on whites. AAs and HAs are thus underrepresented in genetic/genome-wide studies of IR. Uncovering IR-specific genetic signatures in these large minorities may advance the understanding of the biology of IR regulation and further, as cancer biomarkers, improve the prediction ability for CRC risk. It can also promote the development of genetically focused, tailored interventions for CRC preventive and therapeutic efforts.

For this reason, we conducted a genomic study of IR and, with validated IR-specific genetic variants, tested for the association with CRC risk specifically focusing on AA and HA postmenopausal women. Since the allele frequencies of modeled genotypes and their effects on IR and CRC are race/ethnicity specific, we conducted our genomic study separately within AA and HA women. We examined more than 780 IR single-nucleotide polymorphisms (SNPs) that have been detected as top genetic signals in the largest and independent genome-wide association (GWA) studies (20–25). With the IR-SNPs validated in our datasets, we tested for the association with CRC development.

Moreover, although obesity is most prevalent in both AA and HA women of all racial/ethnic groups (26), and the diabetes rates within those 2 minority groups are higher than they are in whites (27), CRC incidence is more prevalent in AA women than in HA women (3, 28). Our preliminary analysis also supported this phenomenon [hazard ratio (HR)_HA *vs*. AA_ = 1.85, 95% confidence interval (CI): 1.08 – 3.18] (**Figure S1**); this suggests the potential role of other lifestyle factors (e.g., diet, smoking, alcohol, female hormones) that are also associated with CRC risk (2, 29–38) in mediating the racial/ethnic differences in CRC risk. Therefore, we incorporated these CRC-associated lifestyle factors with IR genetic markers that we validated for their associations with IR and CRC risk and established risk-prediction models in AA and HA women. By computing the risk prediction for each variable for CRC risk, we detected the most influential genetic markers and lifestyle factors. We next estimated the prediction ability and accuracy of those risk factors, both singly and combined. We further computed to what extent genetic and lifestyle factors, separately and together, influence the development of CRC in each racial/ethnic group [i.e., population attributable risk (PAR)]. Eventually, we estimated an attributable risk (AR) for the common risk factors across the 2 groups to explore potential factors that may play a key role in differentiating the risk for CRC between groups.

## Materials And Methods

### Study Subjects

Our study subjects were AA and HA postmenopausal women who had been enrolled in the SNP Health Association Resource (SHARe), which is a prospective cohort of the minorities as a part of Women’s Health Initiative Database for Genotypes and Phenotypes (WHI dbGaP) Harmonized and Imputed GWA Studies with the aim of revealing genes/genetic variants in association with quantitative traits with enhanced statistical power in those racial/ethnic minorities. Details of the study design and rationale have been described elsewhere (39–41). In brief, healthy women were recruited at 40 WHI-designated clinical centers across the United States from 1993 through 1998 if they were 50–79 years old, postmenopausal, and expected to stay near the clinical centers for at least 3 years after enrollment. Women were excluded if they had any medical conditions associated with predicted survival of less than 3 years in the judgment of the clinical center physician. They had been further enrolled in the WHI dbGaP study if they had met eligibility for data submission to the dbGaP resource and provided DNA samples. Participants provided written informed consent at enrollment. Among 10,818 women (7,470 AA and 3,348 HA) who reported their race or ethnicity as AA or HA, we applied exclusion criteria as follows: genomic data quality control (QC); a history of diabetes; a diagnosis of any cancer type at enrollment; and less than 1-year follow-up. Ultimately, our study cohort contained 6,678 women (4,692 AA and 1,986 HA). After enrollment, they had been followed through August 2014, with a median follow-up of 15 years at the end point. By their last follow-up, 89 women [73 (1.5%) AA and 16 (0.8%) HA] had developed primary CRC. The institutional review boards of the WHI participating clinical centers and the University of California, Los Angeles approved our study.

### Selection of IR SNPs

We employed data to select IR-specific SNPs from the publicly available genomic resource on glycemic traits, the Meta-Analyses of Glucose and Insulin-related traits Consortium (MAGIC; www.magicinvestigators.org) (20–23). MAGIC had analyzed FG and FI as continuous variables. We also used 2 other GWA-based data resources for racial/ethnic minorities. One (24) detected SNPs associated with FG in a 500-kb linkage disequilibrium (LD) block, and the other (25) found functional SNPs for glucose intolerance. Among a total of 1,344 FG-SNPs and 313 FI-SNPs identified in these studies, 689 FG and 91 FI SNPs for AA women and 692 FG and 92 FI SNPs for HA women are available in our SHARe dbGaP study, among which 94 FG and 8 FI SNPs for AAs and 168 FG and 1 FI SNPs for HAs were validated with a relevant phenotype.

### Genotyping and Phenotyping

We extracted genotyping data for the study subjects from the WHI dbGaP SHARe database. Details of genotyping information have been reported (39, 41). DNA samples were obtained from the subject blood samples at baseline and genotyped with Affymetrix 6.0 (Affymetrix, Inc., Santa Clara, CA) at the Fred Hutchinson Cancer Research Center in Seattle, WA. Genomic data were normalized to Genome Reference Consortium Human Build 37, imputed with the 1000 genomes reference panels, and harmonized *via* pairwise concordance among samples across WHI GWA studies. We compared the self-reported ethnicity with genetic principal component (PC). If any discrepancy or admixed participant was found, the subject was labeled as being genetically inconsistent; no one in the SHARe data was identified whose genetic ethnicity was inconsistent. We conducted genomic data QC, filtering out those SNPs with a missing-call rate of ≥ 2%, a Hardy-Weinberg equilibrium of p < 1E–04, and Ř^2^ < 0.6imputation quality (42). Further, we excluded those individuals with unexpected duplicates, first- and second-degree relatives, and outliers defined by our genetic PC analysis.

Blood samples after fasting were derived from each subject at baseline by trained phlebotomists. Serum levels of glucose and insulin were measured using the hexokinase method on a Hitachi 747 instrument (Boehringer Mannheim Diagnostics, Indianapolis, IN) and using a radioimmunoassay method (Linco Research, Inc., St. Louis, MO), respectively, with average coefficients of variation of 1.28% and 10.93%, respectively.

### Lifestyle Factors and Cancer Outcome

To select CRC-associated lifestyle factors, we performed a literature review (2, 29–38, 43–46) particularly focusing on AAs and HAs. On the basis of our review, we extracted the following lifestyle variables from the SHARe database: age at enrollment; family history of CRC (genetic inheritance); lipid metabolic profiles; anthropometric measures (body mass index [BMI], waist circumference, and waist-to-hip ratio); physical activity; alcohol intake (daily dietary alcohol intake and history of alcohol intake); smoking (number of years as a regular smoker and number of cigarettes smoked daily); nutrition (dietary fiber; daily fruits and vegetables; percent calories from protein; percent calories from saturated and mono- and polyunsaturated fatty acids [SFA, MFA, and PFA, respectively]; dietary calcium; vitamin K; and total sugars); age at menopause; and duration of oral contraceptive (OC) use. Additionally, we included in our data analysis the following variables: demographic and socioeconomic variables (education; marital status; and employment); comorbid conditions (depressive symptoms; cardiovascular disease ever; and hypertension ever); and other reproductive histories (age at menarche; number of pregnancies; duration of breast feeding; oophorectomy and/or hysterectomy; and unopposed/opposed exogenous estrogen use). All the aforementioned variables had been obtained at baseline from subjects *via* self-administered questionnaires, except weight, height, and waist/hip circumferences, which had been measured by trained clinical staff. The WHI coordinating clinical centers monitored all the data collection processes. By using those 35 selected variables, we further conducted preliminary univariate and stepwise/multiple regressions in association with CRC risk and checked multicollinearity between variables.

A diagnosis of primary CRC in the study subjects was confirmed *via* a centralized review of medical records and pathology and cytology reports by the WHI committee of physicians, who followed the National Cancer Institute’s Surveillance, Epidemiology, and End-Results guidelines (47). The time between enrollment and CRC diagnosis, censoring, or study end-point was computed, first in days, and then converted to years.

### Statistical Analysis

We conducted linear and Cox proportional hazards regressions to estimate the relationship of GWA-based IR-SNPs with naturally log-transformed FG (mg/dl)/FI (µIU/ml) and with CRC risk, respectively, after confirming that the assumptions for each were met. Both regression analyses were adjusted for age and 10 genetic PCs that account for racial/ethnic ancestry variations. A 2-tailed p < 0.05 for validation tests of FG/FI and association tests with CRC risk was considered nominally significant. After the Bonferroni correction for multiple comparisons, p < 7E-05 for FG, p < 5E-04 for FI, and p < 5E-04 (in AAs) and p < 3E-04 (in HAs) for CRC risk were considered statistically significant.

With those SNPs validated for their association with relevant phenotype and CRC risk and the selected lifestyle factors, we conducted a Random Survival Forest (RSF) analysis. RSF is a tree-based ensemble machine-learning method that accounts for the nonlinear effects and high-order interactions among variables (48); it has outperformed traditional prediction models, successfully yielding more accurate predictions (49–53). The 2 key predictive values generated from the RSF model are minimal depth (MD); those variables with a small MD are highly predictive, and variable importance (VIMP); those variables with a larger VIMP are more predictive (48, 54). RSF creates a tree from the bootstrapped samples by maximizing survival differences across daughter nodes and, by repeating this process numerous times (n = 5,000 trees in this study), generates a forest of trees. Using the out-of-bag (OOB) data, we first computed the prediction error and next, the OOB concordance index (c-index = 1 – prediction error), which is conceptually similar to the area under the receiver operating characteristic (ROC) curve (AUC) (55, 56).

We applied a multimodal RSF approach in the AA and HA groups to detect the most influential predictors for CRC risk among the SNPs and lifestyle factors. In a separate RSF analysis within genetic markers and lifestyle variables, we first compared the 2 key predictive values, MD and VIMP, in the plot. Next, we computed the incremental error rate of each variable within the nested sequenced RSF models. Last, we estimated the drop error rate in each variable ranked by MD in the nested models to detect variables that contribute to reducing the prediction error rate. By using the identified topmost influential SNPs and lifestyle factors, both singly and combined in each group of women, we further estimated the OOB c-index within the nested RSF model and plotted an ROC curve (57) to quantitatively measure their prediction performance. Further, we estimated the combined effect of the topmost genetic and lifestyle predictors on CRC risk using Cox regression in each racial/ethnic group. After a 2-tailed p value was corrected for multiple comparisons *via* the Benjamini-Hochberg method, a 5% false discovery rate (FDR) was considered statistically significant. Eventually, by using the most predictive variables in each group, we computed the PAR percentage (58) to determine the extent to which CRC cases in the group are attributed to genetic and lifestyle factors, singly and in combination. Last, we identified common variables from the most influential variables among the AA and HA women, and by estimating the AR percentage for each variable (59), we explored what variable(s) may contribute to the racial difference in CRC incidence between the groups. Multiple R packages were used (R v4.0.4, pROC survival, survivalROC, randomForestSRC, ggRandomForests, ggplot2, ggthemes, and gamlss).

## Results

Between the 94 FG and 8 FI SNPs in AA women (**Tables S2A, B**) and the 168 FG and 1 FI SNPs (**Tables S3A, S2B**) in HA women, which were validated with a relevant phenotype nominally and after multiple comparison corrections, 35 FG SNPs overlapped, while none of the FI SNPs were shared by the AA and HA groups. In the analysis of those validated SNPs for their association with the risk of CRC development, 10 SNPs in AA women (**Table S2C**) and 27 SNPs in HA women (**Table S3C**) were significant nominally and after multiple comparison correction. Of note, they were all identified among the FG SNPs and were not shared by the 2 groups: the FG SNPs of AAs were from the chromosomes 5 and 7, whereas the FG SNPs of HAs were from chromosome 2. Using those SNPs validated with the phenotype and CRC outcomes in each group of women, we proceeded to the next step, RSF analysis.

### Multimodal RSF Analysis of Validated SNPs and Selected Lifestyle Factors

**Figure 1:**
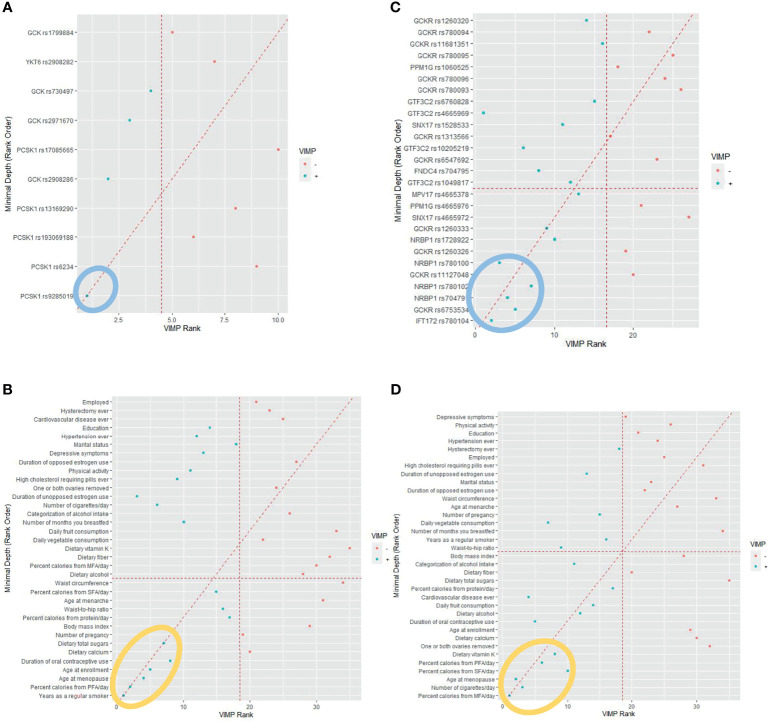
Random survival forest comparing rankings between minimal depth and variable of importance (VIMP). **(A)** African American women. (Note: The 1 genetic marker within the blue oval was identified as the topmost influential predictor. **(B)** African American women. (MFA, monounsaturated fatty acid; PFA, polyunsaturated fatty acid; SFA, saturated fatty acid. Note: The 6 lifestyle variables within the orange oval were identified as the topmost influential predictors. **(C)** Hispanic American women. (Note: The 5 genetic markers within the blue oval were identified as the topmost influential predictors. **(D)** Hispanic American women. (MFA, monounsaturated fatty acid; PFA, polyunsaturated fatty acid; SFA, saturated fatty acid. Note: The 6 lifestyle variables within the orange oval were identified as the topmost influential predictors).

**Figure 2:**
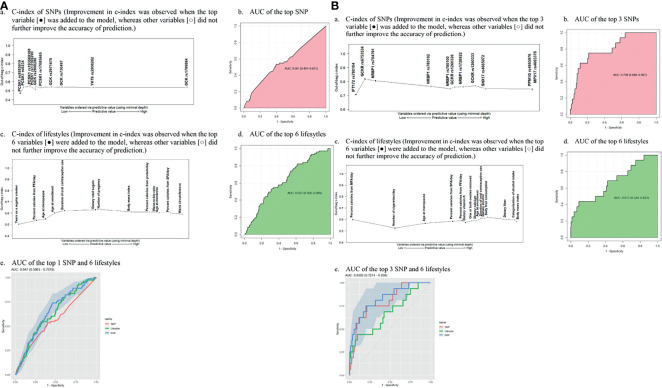
Out-of-bag concordance index (C-index) and area under the receiver operating characteristic curve (AUC) for the topmost genetic and lifestyle factors (MFA, monounsaturated fatty acid; PFA, polyunsaturated fatty acid; SFA, saturated fatty acid SNP; single-nucleotide polymorphism **(A)** African American women. **(a)** C-index of SNPs (Improvement in c-index was observed when the top variable [•] was added to the model, whereas other variables [○] did not further improve the accuracy of prediction. **(b)** AUC of the top SNP **(c)** C-index of lifestyles (Improvement in c-index was observed when the top 6 variables [•] were added to the model, whereas other variables [○] did not further improve the accuracy of prediction.) **(d)** AUC of the top 6 lifesytles **(e)** AUC of the top 1 SNP and 6 lifestyles **(B)**. Hispanic American women. **(a)** C-index of SNPs (Improvement in c-index was observed when the top 3 variable [•] was added to the model, whereas other variables [○] did not further improve the accuracy of prediction.) **(b)** AUC of the top 3 SNPs **(c)**. C-index of lifestyles (Improvement in c-index was observed when the top 6 variables [•] were added to the model, whereas other variables [○] did not further improve the accuracy of prediction.) **(d)** AUC of the top 6 lifestyles **(e)** AUC of the top 3 SNP and 6 lifestyles.

To detect the topmost influential genetic and lifestyle factors in each racial/ethnic group within the RSF prediction model, we adapted a multimodal approach. In separate RSF models within the SNPs and selected lifestyle factors, we first generated a plot of 2 prediction measures, the MD and VIMP (**Figure 1**). In agreement with high ranks between the 2 values in AA women, we detected 1 genetic and 6 lifestyle factors as the topmost predictive variables for CRC risk (**Figures 1A** **, B**): *PCSK1* rs9285019 and years as a regular smoker, percent calories from PFA/day, dietary total sugar intake, age at enrollment, age at menopause, and duration of OC use. Next, we computed the incremental and drop error rates of each SNP and lifestyle variable arranged by MD in the nested sequenced RSF models (**Tables S4A, B**), detecting the same set of the topmost 1 genetic and 6 lifestyle variables, which contributes substantially to reducing the prediction error rate. By using these topmost predictive variables, we further estimated a c-index and AUC (**Table 1**) and plotted them (**Figure 2A**), confirming those top variables’ prediction ability. Specifically, in the c-index plots for the SNP (**Figure 2Aa**) and lifestyles (**Figure 2Ac**), which were ordered by MD rank, those topmost genetic and lifestyle variables were distinctive to improve prediction ability compared with the rest of the variables. The AUC estimations for those topmost genetic and lifestyle variables each presented results similar to those from the c-index estimation (**Figures 2** **Ab, 2Ad**). The combination of the gene- and lifestyle-specific AUC yielded 0.647 (95% CI: 0.587 – 0.708) (**Figure 2** **Ae**), revealing that the topmost lifestyle variables were more substantial contributors to the prediction performance than the top genetic marker was.

**Table 1:** Predictive measures C-index and AUC of the topmost genetic and lifestyle factors in association with colorectal cancer risk.

	African American women			Hispanic American women		
Type of variable	Topmost influential variables*	C-index	AUC (95% CI)	Topmost influential variables*	C-index	AUC (95% CI)
**SNP**	*PCSK1* rs9285019	0.4715	0.561 (0.491 – 0.631)	*IFT172* rs780104	0.7064	0.798 (0.688 – 0.907)
				*GCKR* rs6753534	0.8175	
				*NRBP1* rs704791	0.8048	
**Lifestyle factors**	Years as a regular smoker	0.5023	0.627 (0.566 – 0.689)	% calories from MFA/day	0.5979	0.675 (0.526 – 0.823)
	% calories from PFA/day	0.5356		Number of cigarettes/day	0.5245	
	Age at menopause	0.5486		Age at menopause	0.5655	
	Age at enrollment	0.6014		% calories from SFA/day	0.5836	
	Duration of OC use	0.6223		% calories from PFA/day	0.5896	
	Dietary total sugars	0.6301		Dietary vitamin K	0.5721	
**SNP +** **Lifestyle factors**	1 SNP + 6 lifestyle factors		0.647 (0.586 – 0.708)	3 SNPs + 6 lifestyle factors		0.830 (0.721 – 0.939)

AUC, area under the receiver operating characteristic curve; CI, confidence interval; C-index, concordance index; MFA, monounsaturated fatty acid; OC, oral contraceptive; PFA, polyunsaturated fatty acid; SFA, saturated fatty acid; SNP, single-nucleotide polymorphism.

*Topmost predictive variables were selected on the basis of random survival forest analysis with a multimodal approach.

We applied the same approach to the group of HA women to find their topmost influential variables. We detected 5 SNPs and 6 lifestyles in agreement with high ranks between MD and VIMP (**Figures 1C** **, D**) and, by computing the incremental/drop error rate of each genetic and lifestyle variable (**Tables S4C, D**), we identified those same topmost genetic and lifestyle variables. Due to the high LD (r^2^ > 0.5) within the detected topmost 5 SNPs, we determined 3 SNPs (*IFT172* rs780104, *GCKR* rs6753534, and *NRBP1* rs704791) as the final influential genetic markers and carried them over to the c-index/AUC estimation (**Table 1** and **Figure 2B**). The topmost lifestyle variables identified in the HA women were similar to those detected in the AA women, but more variables were involved: dietary fat intake (SFA/MFA) and dietary vitamin K intake. The c-index and AUC measures from a separate analysis within these topmost SNPs (**Figures 2** **Ba, Bb**) and lifestyle factors (**Figures 2** **Bc, Bd**) also indicated their prediction ability. The AUC from the SNPs and lifestyles together was 0.830 (95% CI 0.721 – 0.939) (**Figure 2** **Be**), in which those top genetic factors contributed more profoundly to the prediction ability than the top lifestyle factors did; this pattern differs from that observed in AA women.

### The Detected Topmost SNPs and Lifestyle Factors: Combined Effects on CRC Risk

**Figure 3:**
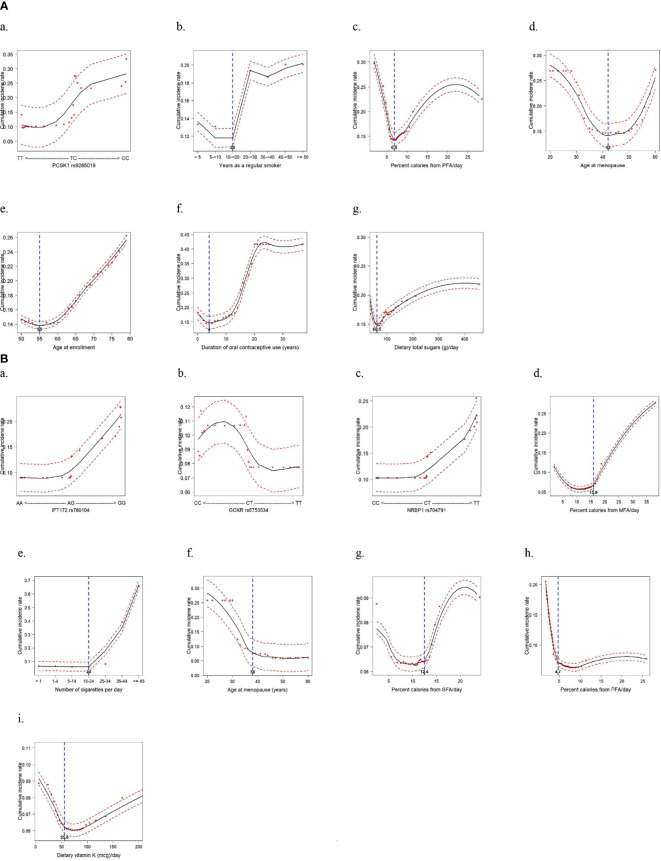
Cumulative incidence rate of colorectal cancer for the topmost predictive genetic and lifestyle variables selected from a random survival forest analysis (Dashed red lines indicate 95% confidence intervals. **(A)** African American women: 1 genetic and 6 lifestyle factors. **(B)** Hispanic American women: 3 genetic and 6 lifestyle factors.

By using the topmost influential IR-SNPs and lifestyle variables in each racial/ethnic group, we implemented the machine-learning process using the RSF model to compute the cumulative predictive CRC incidence rate by adjusting for confounding variables and a nonlinearity effect of the variable on CRC incidence (**Figure 3**). In the AA group, the risk genotype and risk lifestyles were defined according to their cutoff values, which were determined by their risk distribution in the plot: *PCSK1* rs9285019 TC+CC; ≥ 20 years as a regular smoker; ≤ 6.8% of daily calories from PFA; age > 42 years at menopause; age between 56 and 79 years at enrollment; 5–37 years of OC use; and > 60.5 g of total dietary sugar intake. In the HA group, *IFT172* rs780104 GG, *GCKR* rs6753534 CC, and *NRBP1* rs704791 TT were determined to be the risk genotypes. Also, > 15.9% of daily calories from MFA; ≥ 25 cigarettes smoked daily; age ≤ 38 years at menopause; > 12.4% of daily calories from SFA; ≤ 4.7% of daily calories from PFA; and ≤ 55.6 mg of dietary vitamin K were defined as the risk lifestyles. It is noteworthy that in both groups, a greater daily intake of calories from PFA was shown to be a protective factor against CRC development. Interestingly, prolonged exposure to female hormones (i.e., late menopause and/or longer OC use) was revealed to be a risk factor for CRC development among the AA women, but in the HA women it was a protective factor.

Having categorized those topmost SNPs and lifestyle variables accordingly, we first investigated their individual risks for CRC (by adjusting for the others), thus confirming their single effects on CRC risk (**Table S5**). Indeed, the effect magnitude of the individual SNPs was much greater in the HA group than it was in AA group; this corresponded with the finding of the greater influence of those topmost SNPs on the AUC in the HA than in the AA women. Also, whereas most lifestyle variables were not significant after accounting for the others in the AA group, some of the lifestyle variables in the HA group were significant, having a substantial effect on CRC risk.

Next, we tested for the combined effect of the topmost influential SNPs and lifestyle variables, both singly and together, on the risk for CRC. Referring to the analysis of the number of combined lifestyles in relation to CRC risk (**Figure S2A**) in AA women, we combined the AA women with 5 or 6 risk lifestyles and compared their risk with that of the AA women with ≤ 4 risk lifestyles. This yielded an approximately 3 times increased risk for CRC in this high risk–lifestyle group (**Table 2**). Further, we combined the risk genotype and lifestyle factors to test for their synergistic effect on increasing risk for CRC. Compared with the women without either of genetic and lifestyle factors, the AA women with both risk factors were associated with a 4-times higher risk for developing CRC, suggesting a gene–lifestyle dose-response relationship in both additive and multiplicative interaction models (HR of G×E = 1.08). In the HA women, stronger effects of SNPs and lifestyle factors, in each combination, were observed (**Table 3**): about 10 times higher risk for CRC among those with 2 or 3 risk alleles than among those with none or 1 risk allele; and about 7 times greater CRC risk among those with 3 risk lifestyles than among those with ≤ 2 risk lifestyles. The maximum number of lifestyle combinations was 3, and they were categorized on the basis of CRC risk distribution by the number of combined lifestyles (**Figure S2B**). Consistent with our findings of the AA women, the HA women who had both risk genotypes and risk lifestyles had greater and much stronger (> 58 times) risk for CRC than did those who did not have either of them (**Table 3**). This also suggests that the most-predictive genetic and lifestyle factors in combination synergistically increased the predictability of CRC risk in both additive and multiplicative interaction models (HR of G×E = 1.38).

**Table 2:** African American women: combined effect of risk genotypes and risk lifestyles on colorectal cancer risk and population-attributable risk percentage.

Number of risks	n	HR (95% CI)	*p*	PAR (%)^†^
Risk genotypes^£^				
0	2,756	reference		22.9
1	1,936	**1.64 (1.03 – 2.59)**	**0.0356**	
Risk lifestyles ^¶^				
0	3,097	reference		33.6
1	1,595	**2.61 (1.65 – 4.15)**	**4.66E-05**	
Risk genotypes plus lifestyle factors ^§^				
0	1,859	reference		44.9
Risk genotypes only	1,238	1.51 **(**0.75 – 3.02**)**	0.2450	
Risk lifestyles only	897	**2.46 (1.25 – 4.83)**	0.0088*	
Both risks of genotypes and lifestyles	698	**4.02 (2.12 – 7.60)**	1.95E-05*	
*p*_trend_			**1.00E-04**	

CI, confidence interval; HR, hazard ratio; PAR, population attributable risk. Numbers in bold face are statistically significant.

^†^PAR(%) reflects, in total African American women, a risk of colorectal cancer attributable to the risk genotypes and the risk lifestyles, both singly and in combination.

^£^The number of risk genotype (PCSK1 rs9285019 TC+CC) was defined as follows: 0 (none) vs. 1 (1 risk allele).

^¶^The number of lifestyles (≥ 20 years as a regular smoker, ≤ 6.8% of calories from polyunsaturated fatty acid/day, > 42 years old at menopause, 56–79 years old at enrollment, 5–37 years of oral contraceptive use, and > 60.5 g of dietary total sugars) was determined on the basis of analysis for the combined lifestyle factors (**Figure S2A**) and defined as follows: 0 (null/1/2/3/4 risk lifestyles) vs. 1 (5/6 risk lifestyles).

^§^The combined number of risk genotypes and risk lifestyles was based on risk genotype defined as 0 (none) and 1 (1 risk allele), and risk lifestyles defined as 0 (null/1/2/3/4 risk lifestyles) and 1 (5/6 risk lifestyles). The ultimate number of risk genotypes combined with risk lifestyles was defined as 0 (no risk genotypes and risk lifestyles); and risk genotypes (only risk genotypes) and risk lifestyles (only risk lifestyles), separately and together.

*p values with false discovery rate < 0.05 are shown after multiple comparison corrections via the Benjamini-Hochberg method.

**Table 3:** Hispanic American women: combined effect of risk genotypes and risk lifestyles on colorectal cancer risk and population-attributable risk percentage.

Number of risks	n	HR (95% CI)	*p*	PAR (%)^†^
Risk genotypes^£^				
0	1,495	reference		66.8
1	491	**9.57 (3.08 – 29.67)**	**9.20E-05**	
Risk lifestyles ^¶^				
0	1,850	Reference		26.2
1	136	**6.63 (2.30 – 19.11)**	**0.0005**	
Risk genotypes plus lifestyle factors ^§^				
0	1,394	Reference		
Risk genotypes only	456	**8.55 (2.27 – 32.24)**	1.53E-03*	73.3
Risk lifestyles only	101	4.97 (0.52 – 47.76)	0.1653	
Both risks of genotypes and lifestyles	35	**58.76 (13.15 – 262.68)**	9.73E-08*	
*p*_trend_			**2.00E-06**	

CI, confidence interval; HR, hazard ratio; PAR, population attributable risk. Numbers in bold face are statistically significant.

^†^PAR(%) reflects, in total Hispanic African women, a risk of colorectal cancer attributable to the risk genotypes and the risk lifestyles, both singly and in combination.

^£^The number of risk genotypes (IFT172 rs780104 GG; GCKR rs6753534 CC; and NRBP1 rs704791 TT) was defined as follows: 0 (none/1 risk allele) vs. 1 (2/3 risk alleles).

^¶^The maximum combined number of lifestyles (> 15.9% of calories from monounsaturated fatty acid [FA]/day, ≥ 25 cigarettes/day, ≤ 38 years old at menopause, > 12.4% of calories from saturated FA/day, ≤ 4.7% of calories from polyunsaturated FA/day, and ≤ 55.6 mg of dietary vitamin K) was 3. The number of lifestyles was determined on the basis of analysis for the combined lifestyle factors (**Figure S2B**) and defined as follows: 0 (null/1/2 risk lifestyles) vs. 1 (3 risk lifestyles).

^§^The combined number of risk genotypes and risk lifestyles was based on risk genotypes defined as 0 (none/1 risk allele) and 1 (2/3 risk alleles), and risk lifestyles defined as 0 (null/1/2 risk lifestyles) and 1 (3 risk lifestyles). The ultimate number of risk genotypes combined with risk lifestyles was defined as 0 (no risk genotypes and risk lifestyles); and risk genotypes (only risk genotypes) and risk lifestyles (only risk lifestyles), separately and together.

*p values with false discovery rate < 0.05 were shown after multiple comparison corrections via the Benjamini-Hochberg method.

### PAR Percentage for the Combined Topmost Variables in Each Group and AR Percentage for the Variables Common to Both Groups

In the estimation of PAR percentage from the topmost genetic and lifestyle variables in AA women, 23% of their CRC cases were attributed to one top SNP, and 33% were attributed to lifestyle factors in combination. Further, 45% of the CRC cases in AA women were attributed to those genetic and lifestyle factors combined, implicating that almost half of the cases could have been prevented if they would not have had such risk factors (**Table 2**). In HA women, 67% of the CRC cases was attributed to genetic factors, and 26% was attributed to risk lifestyles. When the top genetic and lifestyle factors were combined, about 70% of the CRC cases could have been prevented if they had not possessed such risk factors (**Table 3**).

In addition, we detected 3 common lifestyle factors among the topmost influential markers shared by the AA and HA women: smoking, age at menopause, and daily calorie intake from PFA (**Table 4**). The AR percentages from smoking between the groups were similar, but those from age at menopause and dietary PFA intake were 2 times and 4 times higher, respectively, in the HA than they were in the AA women. The HA women’s long lifetime exposure to female hormones tended to be protective, and the threshold of daily PFA intake to prevent CRC risk was less than the AA women’s (5% *vs*. 7%, respectively). Altogether, we postulate that these 2 lifestyle factors play an important role in mediating the difference in CRC risk between AA and HA women.

**Table 4:** Colorectal cancer attributable risk for the lifestyle factors detected as the topmost predictive variables in both African American and Hispanic American women.

Overlapped variables:the topmost predictors	African American Women	Hispanic American women
	AR (%)	AR (%)
Smoking^†^	61.7	87.4
Age at menopause	28.2	57.1
percent calories from PFA/day	12.7	48.9

AR, attributable risk; PFA, polyunsaturated fatty acid.

^†^The modeled variable for smoking factor is years as a regular smoker in African American women and the number of cigarettes smoked daily in Hispanic American women.

## Discussion

Despite some improvement in healthcare disparities between different racial/ethnic categories in cancer medicine, disparities in cancer genomic science still exist for AA and HA women, the 2 largest minorities of the U.S. population, which are underrepresented in collection, aggregation, and analysis of genomic data for studies of cancer risk factors. Here we focused on AA and HA postmenopausal women to examine genetic markers of IR, one of the main biologic mechanisms of colorectal carcinogenesis, by using an extensive set of GWA-based IR SNPs. In addition to these genetic factors, by incorporating CRC-associated lifestyle variables to establish the CRC risk prediction model for each racial/ethnic group, we detected the topmost influential genetic and lifestyle factors. The combined topmost genetic- and lifestyle-specific markers revealed a synergistic effect on increasing the CRC risk by explaining a considerable portion of their cancer risk. Thus, constructing CRC risk profiles with those topmost markers substantially improved the risk-prediction performance. We believe that these results could be used in the development of genetically focused interventions for cancer prevention and therapeutic effort, and allow progress toward reducing cancer disparity in those minorities.

Most of the topmost FG-SNPs we detected are found in the intronic and intergenic regions of genes that play well-established roles in modulating glucose metabolism, implicating that these genetic variations may influence glucose homeostasis. In AA women, the genetic variant in the *PCSK1* gene was associated with FG concentration as well as increased risk for CRC. The *PCSK1* gene encodes prohormone convertase 1/3, which mediates the cleavage of proinsulin in the process of insulin biosynthesis. Thus, that gene mutation leads to the loss-of-function defect in insulin production, eventually resulting in impaired glucose tolerance (60–63). Further, the mutation of this gene is associated with carcinogenesis and enhanced cancer growth, particularly in the liver metastasis of primary CRC cells (64), suggesting the involvement of the convertases in the selective process of liver metastasis. To the best of our knowledge, ours is the first report of the *PCSK1* gene variation’s association with primary CRC risk, particularly in AA women.

Of the topmost FG-SNPs detected in HA women, the genetic variant of *GCKR* was associated with a higher FG concentration and increased CRC risk. The *GCKR* regulates the activity of glucokinase in liver and pancreatic islet cells (65). For example, when circulating glucose level is low, *GCKR* forms an inactive complex with glucokinase, inhibiting glycolysis (66). Thus, a high degree of inhibition of this enzyme by *GCKR* can result in high FG levels. The genetic variation of *GCKR* in association with FG concentrations was previously reported in AAs (24) but not in HAs. Also, the *GCKR* variation has been associated with the risk of pancreatic cancer (67) and the prognosis of metastatic gastric cancer (68), but no published study so far has examined its association with CRC risk. Therefore, our findings of FG and CRC risk in HA women are meaningful and warrant replication in further studies with independent datasets. In addition, *NRBP1*, which encodes multidomain putative adapter proteins (69), has an anti-tumor role against CRC tumorigenesis and progression, as an *in vivo*/*in vitro* study (70) showed that the higher expression of *NRBP1* inhibited CRC cell proliferation and anti-apoptosis and correlated with better prognosis. *NRBP1* regulates the apoptotic pathway by inhibiting *Jab1*-mediated *JNK* signaling, which is essential in gene translation and regulation of cellular apoptosis (70–72); it may thus play a key role in suppressing CRC tumorigenesis. Supported by these earlier findings, our study reported that the variation of the *NRBP1* gene increased the risk of CRC, specifically in HA women. Last, the genetic variants of *IFT172* that encodes a subunit of the intraflagellar transport subcomplex *IFT-B*, which is necessary for ciliary assembly and maintenance, have been associated with ulcerative colitis and Crohn’s disease (73), but their associations with CRC risk, as detected in our study, have not been previously reported, warranting future replication studies.

Among the 3 topmost influential factors shared by the AA and HA groups, the effect of smoking on CRC risk was strongest in both groups. As revealed in a recent Mendelian randomization study (31), prolonged lifetime exposure to cigarette smoking is positively associated with CRC risk. The carcinogens emitted by tobacco smoke into the digestive system and bloodstream promote tumorigenesis in colorectal mucosa (74). In particular, AA individuals tend to have higher total equivalents of nicotine per number of cigarettes smoked daily than individuals of other racial/ethnic groups, and their CRC screening rate is lower in active smokers than in never smokers (75); thus, screening in the high-risk group (active/longer-term regular smokers) is strongly recommended.

Both groups in our study had greater risk for CRC when they had lower daily intake of PFAs. Previous studies (29, 76) support our finding, by reporting that the decreased proportions of red blood cell PFAs and less intake of PFAs were associated with increased CRC incidence. PFAs have been shown to suppress pro-inflammatory cytokine production (77) and reduce triglycerides and low-density lipoprotein particles (78), which are key mediators in carcinogenesis. In our HA women, the CRC risk attributable to low PFA intake was more substantial than it was in our AA women. However, the HA women had a lower threshold of daily PFA intake than AA women in preventing CRC development. Altogether, the effect of less strict requirement of PFA intake in HA women may override their more sensitive influence of low PFA intake on CRC risk and thus, contribute to the lower CRC incidence in HA than in AA women.

Further, older age at menopause is an important risk factor for CRC development in postmenopausal women (79–81), suggesting that longer lifetime exposure to endogenous estrogen may increase the CRC risk. However, in our analysis of HA women, their longer-term exposure to female hormones tended to be protective against CRC risk, even after adjusting for a history of oophorectomy; this suggests a follow-up functional mechanism study in this racial/ethnic subpopulation. Similar to that of PFA intake, this protective role of prolonged lifetime exposure to female hormones in HA women may outweigh the greater effect of short-term hormone exposure on CRC risk than AA women had, explaining in part their lower CRC incidence compared with that of AA women.

Our data on smoking were self-reported, so our results may have been subject to misclassification bias. However, a previous study found high reliability of self-reported assessment of active smoking (82). Also, our RSF analysis may overfit the model with multiple tasks, warranting the conduct of replication studies with independent datasets. We examined AA and HA postmenopausal women, so our findings may not be generalizable to other racial/ethnic populations.

Overall, our study indicates that GWA-level IR SNPs combined with the lifestyle factors of smoking, lifetime exposure to endogenous female hormones, and dietary fat intake synergistically increased the risk for CRC, and the prediction ability and accuracy of these factors was notable. Of those risk factors, dietary intake of PFAs and lifelong exposure to female hormones may play a key role in mediating the racial disparity of CRC risk between AA and HA women. Our findings may improve CRC risk–prediction performance in these medically and scientifically underrepresented subpopulations, and by emphasizing the promotion of genetically informed preventive interventions (e.g., smoking cessation, higher PFA intake) and encouraging CRC screening of individuals who are at high risk owing to particular risk genotypes and behavioral patterns, our results may contribute to reduced cancer disparity in those minorities.

## Data Availability Statement

Publicly available datasets were analyzed in this study. This data can be found here: The data that support the findings of this study are available in accordance with policies developed by the NHLBI and WHI in order to protect sensitive participant information and approved by the Fred Hutchinson Cancer Research Center, which currently serves as the IRB of record for the WHI. Data requests may be made by emailing helpdesk@WHI.org.

## Ethics Statement

The studies involving human participants were reviewed and approved by the institutional review boards of each participating clinical center of the WHI and the University of California, Los Angeles. The patients/participants provided their written informed consent to participate in this study.

## Author Contributions

SJ, ES, MP, HY, and JP designed the study. SJ performed the genomic data QC. SJ performed the statistical analysis and SJ, ES, MP, HY, and JP interpreted the data. JP and ES supervised the genomic data QC and analysis and participated in the study coordination. JP oversaw the project. SJ secured funding for this project. All participated in writing and editing the paper. All authors contributed to the article and approved the submitted version.

## Funding

This study was supported by the National Institute of Nursing Research of the National Institutes of Health under Award Number K01NR017852.

## Conflict of Interest

The authors declare that the research was conducted in the absence of any commercial or financial relationships that could be construed as a potential conflict of interest.

The handling editor declared a shared affiliation, though no other collaboration, with several of the authors SJ, ES, MP, JP.

## Publisher’s Note

All claims expressed in this article are solely those of the authors and do not necessarily represent those of their affiliated organizations, or those of the publisher, the editors and the reviewers. Any product that may be evaluated in this article, or claim that may be made by its manufacturer, is not guaranteed or endorsed by the publisher.
