# Supplementary material for: Synergistic Effects of Genetic Variants of Glucose Homeostasis and Lifelong Exposures to Cigarette Smoking, Female Hormones, and Dietary Fat Intake on Primary Colorectal Cancer Development in African and Hispanic/Latino American Women
Source: Front Oncol. 2021 Oct 7;11:760243. doi: 10.3389/fonc.2021.760243 (PMC8529283; doi:10.3389/fonc.2021.760243)

Figure S1. Racial difference in colorectal incidence rate between Hispanic American (HA) and African American (AA) women (CI, confidence interval, HR, hazard ratio)


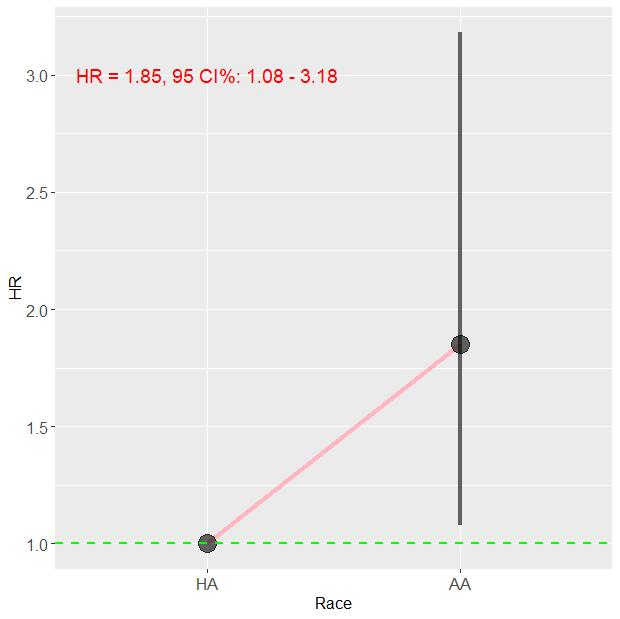

Supplement: Supplementary file 1 [file DataSheet_1.zip › Figure S1.HR for CRC.docx]
