# Supplementary material for: Synergistic Effects of Genetic Variants of Glucose Homeostasis and Lifelong Exposures to Cigarette Smoking, Female Hormones, and Dietary Fat Intake on Primary Colorectal Cancer Development in African and Hispanic/Latino American Women
Source: Front Oncol. 2021 Oct 7;11:760243. doi: 10.3389/fonc.2021.760243 (PMC8529283; doi:10.3389/fonc.2021.760243)

Figure S2. Number of combined risk lifestyles and the corresponding hazard ratio for a risk of colorectal cancer.

1. African American women: of the 6 combined lifestyles, 4 or fewer risk lifestyles within an orange oval were categorized for further analysis of combined genetic and lifestyle factors


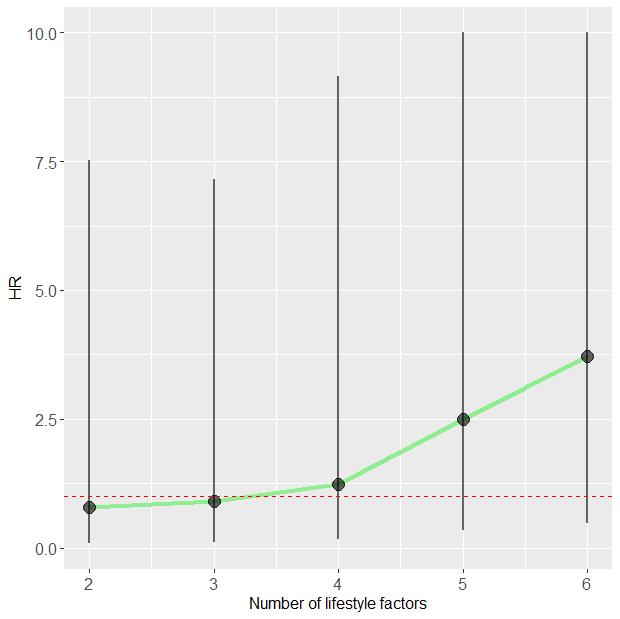


1. Hispanic American women: of the 3 combined lifestyles, 2 or fewer risk lifestyles within an orange oval were categorized for further analysis of combined genetic and lifestyle factors


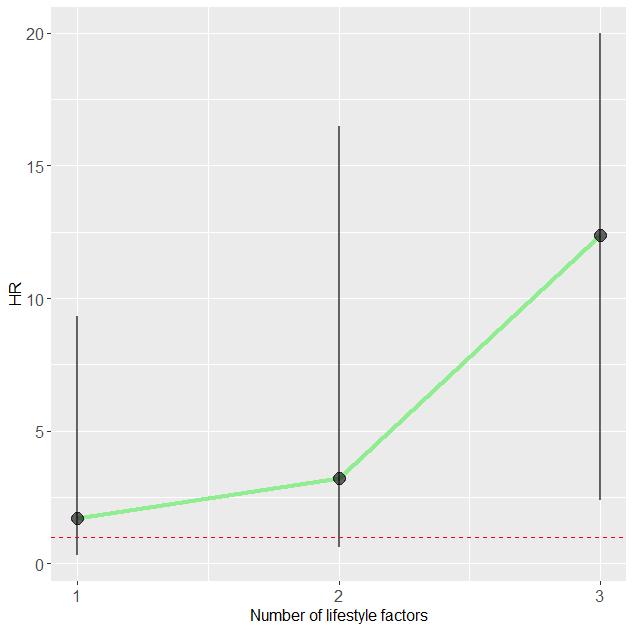

Supplement: Supplementary file 1 [file DataSheet_1.zip › Figure S2.HR for beh group.docx]
