# Supplementary material for: Synergistic Effects of Genetic Variants of Glucose Homeostasis and Lifelong Exposures to Cigarette Smoking, Female Hormones, and Dietary Fat Intake on Primary Colorectal Cancer Development in African and Hispanic/Latino American Women
Source: Front Oncol. 2021 Oct 7;11:760243. doi: 10.3389/fonc.2021.760243 (PMC8529283; doi:10.3389/fonc.2021.760243)
Supplement: Supplementary file 1 [file DataSheet_1.zip › Table S1.AA.HA.ass with IR.obes.docx]

Table S1. Cox regression analyses of the fasting levels of glucose and insulin and obesity variables predicting colorectal cancer risk in African American and Hispanic American postmenopausal women

|  | **African American women** | |  | **Hispanic American women** | |
| --- | --- | --- | --- | --- | --- |
|  | **HR (95% CI)** | **p** |  | **HR (95% CI)** | **p** |
| **Blood concentration** |  |  |  |  |  |
| Fasting glucose (mg/dl)* | 1.61 (0.31 – 8.31) | 0.573 |  | 19.58 (1.68 – 227.8) | 0.0175 |
| Fasting insulin (µIU/ml)* | 1.002 (0.68 – 1.47) | 0.993 |  | 1.21 (0.53 – 2.73) | 0.650 |
|  |  |  |  |  |  |
| **Obesity variable** |  |  |  |  |  |
| Waist-to-hip ratio** | 1.29 (0.05 – 32.48) | 0.876 |  | 19.68 (0.04 – 8808) | 0.339 |
| Waist-to-hip ratio |  |  |  |  |  |
| ≤ 0.85 | reference |  |  | reference |  |
| > 0.85 | 1.21 (0.73 – 1.99) | 0.465 |  | 1.38 (0.48 – 3.98) | 0.549 |
| Waist circumference |  |  |  |  |  |
| ≤ 88 cm | reference |  |  | N/A | N/A |
| > 88 cm | 1.23 (0.77 – 1.94) | 0.386 |  | N/A | N/A |

CI, confidence interval; HR, hazard ratio; N/A, not available.

* The fasting concentrations were naturally log-transformed for the analysis.

** The waist-to-hip ratio was estimated as the ratio of the waist circumference (cm) to the hip circumference (cm) and analyzed as a continuous variable.
