# Supplementary material for: Synergistic Effects of Genetic Variants of Glucose Homeostasis and Lifelong Exposures to Cigarette Smoking, Female Hormones, and Dietary Fat Intake on Primary Colorectal Cancer Development in African and Hispanic/Latino American Women
Source: Front Oncol. 2021 Oct 7;11:760243. doi: 10.3389/fonc.2021.760243 (PMC8529283; doi:10.3389/fonc.2021.760243)
Supplement: Supplementary file 1 [file DataSheet_1.zip › Table S2.AA.SNP-FG.FI.CRC.docx]

Table S2. African American women: FG/FI-SNPs in association with colorectal cancer risk

1. Among the GWA FG-SNPs derived from previous GWA studies, 94 SNPs, nominally and after multiple comparison correction, were associated with the fasting level of naturally log-transformed glucose in the WHI SHARe data.

| **SNP** | **Chr** | **Position¥** | **Allele** | | **Alt Allele Frequency** | **Beta¶** | **SE** | ***p*** |
| --- | --- | --- | --- | --- | --- | --- | --- | --- |
|  |  |  | **Ref** | **Alt** |  |  |  |  |
| rs11264743 | 1 | 153941514 | T | C | 0.865 | 0.008 | 0.0041 | 0.0393 |
| rs1260326* | 2 | 27730940 | C | T | 0.162 | 0.008 | 0.0038 | 0.0404 |
| rs780094* | 2 | 27741237 | C | T | 0.186 | 0.008 | 0.0035 | 0.0196 |
| rs780093* | 2 | 27742603 | T | C | 0.835 | -0.008 | 0.0037 | 0.0312 |
| rs6753534 | 2 | 27752871 | T | C | 0.360 | 0.006 | 0.0030 | 0.0370 |
| rs4665985* | 2 | 27753878 | C | A | 0.882 | -0.010 | 0.0048 | 0.0312 |
| rs11678685 | 2 | 169710607 | A | G | 0.805 | 0.009 | 0.0035 | 0.0070 |
| rs34177044* | 2 | 169754485 | A | G | 0.740 | 0.007 | 0.0033 | 0.0446 |
| rs1402837* | 2 | 169757354 | T | C | 0.695 | 0.007 | 0.0030 | 0.0160 |
| rs560887* | 2 | 169763148 | T | C | 0.931 | -0.016 | 0.0057 | 0.0039 |
| rs17539351 | 2 | 169766560 | C | T | 0.023 | 0.021 | 0.0105 | 0.0471 |
| rs475612 | 2 | 169776746 | T | C | 0.809 | -0.008 | 0.0036 | 0.0311 |
| rs518598 | 2 | 169776914 | C | T | 0.185 | 0.008 | 0.0036 | 0.0294 |
| rs557462* | 2 | 169777595 | C | T | 0.921 | -0.015 | 0.0053 | 0.0037 |
| rs569829* | 2 | 169782869 | T | C | 0.919 | -0.016 | 0.0052 | 0.0020 |
| rs508506* | 2 | 169784955 | A | C | 0.892 | -0.009 | 0.0045 | 0.0485 |
| rs853789* | 2 | 169801488 | A | G | 0.883 | -0.011 | 0.0044 | 0.0144 |
| rs853777* | 2 | 169812217 | C | T | 0.089 | 0.017 | 0.0052 | 0.0009 |
| rs11708067 | 3 | 123065778 | A | G | 0.158 | 0.014 | 0.0037 | 0.0002 |
| rs7613951 | 3 | 123070517 | T | C | 0.876 | -0.014 | 0.0041 | 0.0009 |
| rs11717195 | 3 | 123082398 | C | T | 0.877 | -0.013 | 0.0041 | 0.0012 |
| rs6798189 | 3 | 123095312 | A | G | 0.873 | -0.014 | 0.0041 | 0.0004 |
| rs10934647 | 3 | 123130684 | C | T | 0.123 | 0.011 | 0.0042 | 0.0063 |
| rs17361324 | 3 | 123131254 | T | C | 0.877 | -0.011 | 0.0042 | 0.0064 |
| rs4869272* | 5 | 95539448 | C | T | 0.779 | -0.007 | 0.0033 | 0.0306 |
| rs13179048* | 5 | 95542726 | A | C | 0.904 | -0.009 | 0.0047 | 0.0492 |
| rs12186664* | 5 | 95630225 | T | A | 0.794 | -0.009 | 0.0034 | 0.0077 |
| rs17085593 | 5 | 95630705 | C | G | 0.111 | 0.013 | 0.0044 | 0.0037 |
| rs13162665* | 5 | 95642519 | G | T | 0.904 | -0.011 | 0.0047 | 0.0171 |
| rs10040098 | 5 | 95674266 | C | T | 0.230 | 0.011 | 0.0033 | 0.0009 |
| rs144489757 | 5 | 95694609 | G | C | 0.901 | -0.011 | 0.0047 | 0.0210 |
| rs6863434 | 5 | 95695673 | T | C | 0.665 | -0.007 | 0.0030 | 0.0193 |
| rs1820177 | 5 | 95696429 | T | C | 0.655 | -0.007 | 0.0030 | 0.0227 |
| rs1820176 | 5 | 95696585 | C | T | 0.667 | -0.007 | 0.0030 | 0.0239 |
| rs34874677 | 5 | 95701782 | C | T | 0.714 | -0.010 | 0.0032 | 0.0025 |
| rs6876986 | 5 | 95703329 | C | G | 0.763 | -0.010 | 0.0033 | 0.0024 |
| rs10476552 | 5 | 95709132 | C | T | 0.773 | -0.010 | 0.0033 | 0.0016 |
| rs10036439 | 5 | 95710368 | C | G | 0.666 | -0.007 | 0.0030 | 0.0256 |

Table S2A (Continued)

| **SNP** | **Chr** | **Position¥** | **Allele** | | **Alt Allele Frequency** | **Beta¶** | **SE** | ***p*** |
| --- | --- | --- | --- | --- | --- | --- | --- | --- |
|  |  |  | **Ref** | **Alt** |  |  |  |  |
| rs6869253 | 5 | 95710904 | C | A | 0.766 | -0.010 | 0.0033 | 0.0023 |
| rs7722200 | 5 | 95717440 | T | C | 0.231 | 0.010 | 0.0032 | 0.0032 |
| rs9285019 | 5 | 95719294 | T | C | 0.764 | -0.009 | 0.0032 | 0.0087 |
| rs10213965 | 5 | 95719667 | C | G | 0.769 | -0.009 | 0.0033 | 0.0065 |
| rs10213823 | 5 | 95719676 | C | T | 0.731 | -0.008 | 0.0031 | 0.0071 |
| rs17085655 | 5 | 95719804 | G | A | 0.731 | -0.008 | 0.0031 | 0.0071 |
| rs17085658 | 5 | 95719923 | G | A | 0.731 | -0.008 | 0.0031 | 0.0072 |
| rs7700417 | 5 | 95720359 | C | T | 0.768 | -0.009 | 0.0032 | 0.0038 |
| rs17085665 | 5 | 95723381 | A | G | 0.837 | -0.007 | 0.0038 | 0.0484 |
| rs6234 | 5 | 95728974 | C | G | 0.800 | -0.007 | 0.0035 | 0.0491 |
| rs13169290 | 5 | 95729406 | G | A | 0.161 | 0.008 | 0.0038 | 0.0429 |
| rs193069188 | 5 | 95736557 | C | T | 0.467 | 0.006 | 0.0028 | 0.0408 |
| rs76323047* | 7 | 44185955 | G | A | 0.940 | 0.021 | 0.0066 | 0.0013 |
| rs2908294 | 7 | 44204426 | T | C | 0.859 | 0.014 | 0.0040 | 0.0005 |
| rs2971672 | 7 | 44205906 | C | A | 0.436 | 0.007 | 0.0029 | 0.0214 |
| rs2908293 | 7 | 44209322 | A | G | 0.859 | 0.014 | 0.0040 | 0.0005 |
| rs2908292 | 7 | 44210710 | T | C | 0.807 | 0.011 | 0.0037 | 0.0027 |
| rs2971671 | 7 | 44211337 | C | T | 0.803 | 0.010 | 0.0037 | 0.0056 |
| rs2908290 | 7 | 44216137 | A | G | 0.470 | 0.007 | 0.0028 | 0.0215 |
| rs2300584 | 7 | 44219338 | G | A | 0.861 | 0.012 | 0.0039 | 0.0016 |
| rs10259649 | 7 | 44219705 | C | T | 0.861 | 0.012 | 0.0039 | 0.0016 |
| rs730497* | 7 | 44223721 | A | G | 0.821 | 0.015 | 0.0035 | 3.76E-05**†** |
| rs2908289* | 7 | 44223942 | A | G | 0.748 | 0.007 | 0.0031 | 0.0173 |
| rs2971670 | 7 | 44226101 | T | C | 0.828 | 0.014 | 0.0036 | 6.04E-05**†** |
| rs1799884 | 7 | 44229068 | T | C | 0.828 | 0.014 | 0.0036 | 6.68E-05**†** |
| rs6975024* | 7 | 44231886 | C | T | 0.893 | 0.016 | 0.0047 | 0.0007 |
| rs741037* | 7 | 44232833 | A | G | 0.744 | 0.007 | 0.0031 | 0.0179 |
| rs2908286* | 7 | 44234737 | T | C | 0.827 | 0.015 | 0.0036 | 4.80E-05**†** |
| rs4607517* | 7 | 44235668 | A | G | 0.891 | 0.016 | 0.0047 | 0.0006 |
| rs12056308* | 7 | 44239034 | A | G | 0.732 | 0.007 | 0.0032 | 0.0215 |
| rs1985469* | 7 | 44240324 | T | A | 0.741 | 0.007 | 0.0032 | 0.0176 |
| rs1004558* | 7 | 44240407 | T | C | 0.729 | 0.007 | 0.0032 | 0.0180 |
| rs2971668* | 7 | 44243438 | C | G | 0.753 | 0.010 | 0.0032 | 0.0025 |
| rs2971667* | 7 | 44245060 | C | T | 0.728 | 0.009 | 0.0032 | 0.0048 |
| rs917793* | 7 | 44245853 | T | A | 0.741 | 0.010 | 0.0032 | 0.0030 |
| rs2908282* | 7 | 44248828 | A | G | 0.772 | 0.009 | 0.0032 | 0.0035 |
| rs10885117 | 10 | 113022555 | C | T | 0.361 | -0.006 | 0.0029 | 0.0496 |
| rs10509937 | 10 | 113029061 | C | A | 0.361 | -0.006 | 0.0029 | 0.0494 |
| rs4548554 | 10 | 113029377 | G | A | 0.637 | 0.006 | 0.0029 | 0.0471 |
| rs10787316 | 10 | 113032991 | G | A | 0.384 | -0.006 | 0.0029 | 0.0490 |

Table S2A (Continued)

| **SNP** | **Chr** | **Position¥** | **Allele** | | **Alt Allele Frequency** | **Beta¶** | **SE** | ***p*** |
| --- | --- | --- | --- | --- | --- | --- | --- | --- |
|  |  |  | **Ref** | **Alt** |  |  |  |  |
| rs174537 | 11 | 61552680 | G | T | 0.098 | 0.010 | 0.0048 | 0.0442 |
| rs174546 | 11 | 61569830 | C | T | 0.095 | 0.010 | 0.0047 | 0.0335 |
| rs174547 | 11 | 61570783 | C | T | 0.905 | -0.010 | 0.0047 | 0.0335 |
| rs174549 | 11 | 61571382 | A | G | 0.913 | -0.011 | 0.0049 | 0.0317 |
| rs174550 | 11 | 61571478 | C | T | 0.905 | -0.010 | 0.0047 | 0.0335 |
| rs174555 | 11 | 61579760 | T | C | 0.087 | 0.011 | 0.0049 | 0.0315 |
| rs1535 | 11 | 61597972 | A | G | 0.142 | 0.009 | 0.0041 | 0.0295 |
| rs12222793 | 11 | 92667047 | G | A | 0.609 | 0.006 | 0.0028 | 0.0245 |
| rs11020107 | 11 | 92667730 | C | G | 0.624 | 0.007 | 0.0028 | 0.0077 |
| rs12792753* | 11 | 92668975 | C | T | 0.584 | 0.006 | 0.0027 | 0.0354 |
| rs10830961 | 11 | 92694757 | A | G | 0.744 | -0.007 | 0.0034 | 0.0453 |
| rs10466351* | 11 | 92697981 | T | C | 0.292 | 0.007 | 0.0031 | 0.0211 |
| rs7941837* | 11 | 92699666 | A | T | 0.794 | -0.007 | 0.0035 | 0.0460 |
| rs7945617* | 11 | 92700287 | T | C | 0.797 | -0.007 | 0.0035 | 0.0401 |
| rs10830963* | 11 | 92708710 | G | C | 0.925 | 0.022 | 0.0053 | 3.87E-05**†** |
| rs954750 | 13 | 28463938 | T | C | 0.827 | 0.008 | 0.0040 | 0.0354 |

Alt, alternative; Chr, chromosome; FG, fasting glucose; GWA, genome-wide association; Ref, reference; SE, standard error; SHARe, SNP Health Association Resource; SNP, single-nucleotide polymorphism; WHI, Women’s Health Initiative.

¥ GRCh 37 coordinated.

¶ Beta from regression analysis was adjusted for 10 genetic principal components as well as age.

* SNPs shared by Hispanic/Latino American women are indicated.

† *p* value indicates that the corresponding SNP is statistically significant after the Bonferroni correction for multiple comparisons.

1. Among the GWA FI-SNPs derived from previous GWA studies, 8 SNPs, nominally and after multiple comparison correction, were associated with the fasting level of naturally log-transformed insulin in the WHI SHARe data.

| **SNP** | **Chr** | **Position¥** | **Allele** | | **Alt Allele Frequency** | **Beta¶** | **SE** | ***p*** |
| --- | --- | --- | --- | --- | --- | --- | --- | --- |
|  |  |  | **Ref** | **Alt** |  |  |  |  |
| rs1260326 | 2 | 27730940 | C | T | 0.162 | 0.058 | 0.0169 | 0.0007 |
| rs780094 | 2 | 27741237 | C | T | 0.186 | 0.056 | 0.0156 | 0.0003**†** |
| rs780093 | 2 | 27742603 | T | C | 0.835 | -0.052 | 0.0165 | 0.0015 |
| rs12521454 | 5 | 53297304 | C | T | 0.717 | 0.034 | 0.0134 | 0.0111 |
| rs7735253 | 5 | 53297611 | A | G | 0.717 | 0.034 | 0.0134 | 0.0105 |
| rs6450176 | 5 | 53298025 | A | G | 0.716 | 0.034 | 0.0134 | 0.0103 |
| rs4311394 | 5 | 53300662 | G | A | 0.715 | 0.034 | 0.0134 | 0.0103 |
| rs459193 | 5 | 55806751 | G | A | 0.419 | 0.034 | 0.0123 | 0.0058 |

Alt, alternative; Chr, chromosome; FI, fasting insulin; GWA, genome-wide association; Ref, reference; SE, standard error; SHARe, SNP Health Association Resource; SNP, single-nucleotide polymorphism; WHI, Women’s Health Initiative.

¥ GRCh 37 coordinated.

¶ Beta from regression analysis was adjusted for 10 genetic principal components as well as age.

† *p* value indicates that the corresponding SNP is statistically significant after the Bonferroni correction for multiple comparisons.

1. Cox regressions of 10 genome-wide SNPs associated with FG predicting CRC risk.

|  |  |  | **Allele** | |  |  |  |
| --- | --- | --- | --- | --- | --- | --- | --- |
| **SNP** | **Chr** | **Position¥** | **Ref** | **Alt** | **HR¶ (95% CI)** | **SE** | ***p*** |
| rs9285019 | 5 | 95719294 | T | C | 1.439 (1.011 - 2.048) | 0.1802 | 0.0436 |
| rs17085665 | 5 | 95723381 | A | G | 1.550 (1.044 - 2.303) | 0.2019 | 0.0299 |
| rs6234 | 5 | 95728974 | C | G | 1.584 (1.094 - 2.293) | 0.1887 | 0.0148 |
| rs13169290 | 5 | 95729406 | A | G | 1.595 (1.075 - 2.366) | 0.2012 | 0.0204 |
| rs193069188 | 5 | 95736557 | T | C | 1.436 (1.029 - 2.003) | 0.1699 | 0.0333 |
| rs730497* | 7 | 44223721 | A | G | 1.602 (1.107 - 2.319) | 0.1886 | 0.0124 |
| rs2971670* | 7 | 44226101 | T | C | 1.532 (1.049 - 2.239) | 0.1934 | 0.0274 |
| rs1799884* | 7 | 44229068 | T | C | 1.526 (1.045 - 2.229) | 0.1933 | 0.0288 |
| rs2908286* | 7 | 44234737 | T | C | 1.525 (1.041 - 2.233) | 0.1947 | 0.0303 |
| rs2908282 | 7 | 44248828 | A | G | 1.433 (1.006 - 2.042) | 0.1806 | 0.0462 |

Alt, alternative; Chr, chromosome; CI, confidence interval; CRC, colorectal cancer; FG, fasting glucose; HR, hazard ratio; Ref, reference; SE, standard error; SNP, single-nucleotide polymorphism.

¥ GRCh 37 coordinated.

¶ HR from regression analysis was adjusted for 10 genetic principal components as well as age.

* SNP that is statistically significant after the Bonferroni correction for multiple comparisons.
