# Supplementary material for: Synergistic Effects of Genetic Variants of Glucose Homeostasis and Lifelong Exposures to Cigarette Smoking, Female Hormones, and Dietary Fat Intake on Primary Colorectal Cancer Development in African and Hispanic/Latino American Women
Source: Front Oncol. 2021 Oct 7;11:760243. doi: 10.3389/fonc.2021.760243 (PMC8529283; doi:10.3389/fonc.2021.760243)
Supplement: Supplementary file 1 [file DataSheet_1.zip › Table S3.HA.SNP-FG.FI.CRC.docx]

Table S3. Hispanic American women: FG/FI-SNPs in association with colorectal cancer risk

1. Among the GWA FG-SNPs derived from previous GWA studies, 168 SNPs nominally were associated with the fasting level of naturally log-transformed glucose in the WHI SHARe data.

| **SNP** | **Chr** | **Position¥** | **Allele** | | **Alt Allele Frequency** | **Beta¶** | **SE** | ***p*** |
| --- | --- | --- | --- | --- | --- | --- | --- | --- |
|  |  |  | **Ref** | **Alt** |  |  |  |  |
| rs4665378 | 2 | 27548038 | C | A | 0.511 | 0.008 | 0.0039 | 0.0470 |
| rs1049817 | 2 | 27550967 | G | A | 0.511 | 0.008 | 0.0039 | 0.0447 |
| rs10205219 | 2 | 27568565 | C | T | 0.513 | 0.009 | 0.0039 | 0.0284 |
| rs4665969 | 2 | 27574953 | C | T | 0.527 | 0.008 | 0.0039 | 0.0369 |
| rs6760828 | 2 | 27579231 | C | T | 0.509 | 0.009 | 0.0039 | 0.0192 |
| rs1528533 | 2 | 27595756 | C | G | 0.509 | 0.008 | 0.0039 | 0.0285 |
| rs4665972 | 2 | 27598097 | T | C | 0.644 | -0.012 | 0.0043 | 0.0051 |
| rs1060525 | 2 | 27635582 | G | A | 0.504 | 0.008 | 0.0039 | 0.0352 |
| rs4665976 | 2 | 27640325 | G | A | 0.506 | 0.008 | 0.0039 | 0.0322 |
| rs1728922 | 2 | 27644464 | C | A | 0.507 | 0.008 | 0.0039 | 0.0361 |
| rs780100 | 2 | 27652153 | T | G | 0.507 | 0.008 | 0.0039 | 0.0330 |
| rs704791 | 2 | 27657167 | C | T | 0.507 | 0.008 | 0.0039 | 0.0325 |
| rs780102 | 2 | 27659491 | C | T | 0.507 | 0.008 | 0.0039 | 0.0327 |
| rs780104 | 2 | 27677691 | A | G | 0.507 | 0.008 | 0.0039 | 0.0324 |
| rs704795 | 2 | 27716494 | A | G | 0.510 | 0.008 | 0.0039 | 0.0427 |
| rs1260320 | 2 | 27722416 | A | G | 0.512 | 0.009 | 0.0039 | 0.0255 |
| rs1260326* | 2 | 27730940 | T | C | 0.636 | -0.011 | 0.0041 | 0.0050 |
| rs6547692 | 2 | 27734972 | G | A | 0.584 | -0.013 | 0.0040 | 0.0015 |
| rs780096 | 2 | 27741072 | C | G | 0.578 | -0.013 | 0.0039 | 0.0010 |
| rs780095 | 2 | 27741105 | A | G | 0.585 | -0.013 | 0.0039 | 0.0012 |
| rs780094* | 2 | 27741237 | T | C | 0.632 | -0.011 | 0.0040 | 0.0074 |
| rs780093* | 2 | 27742603 | T | C | 0.634 | -0.011 | 0.0040 | 0.0071 |
| rs11681351 | 2 | 27743423 | A | G | 0.535 | 0.009 | 0.0039 | 0.0273 |
| rs1260333 | 2 | 27748624 | A | G | 0.579 | -0.012 | 0.0039 | 0.0019 |
| rs1313566 | 2 | 27748904 | G | A | 0.579 | -0.012 | 0.0039 | 0.0017 |
| rs11127048 | 2 | 27752463 | G | A | 0.586 | -0.012 | 0.0044 | 0.0082 |
| rs6753534* | 2 | 27752871 | C | T | 0.581 | -0.012 | 0.0042 | 0.0054 |
| rs13431652 | 2 | 169753415 | C | T | 0.790 | -0.013 | 0.0052 | 0.0138 |
| rs34177044* | 2 | 169754485 | A | G | 0.808 | 0.013 | 0.0051 | 0.0115 |
| rs1402837* | 2 | 169757354 | T | C | 0.802 | 0.013 | 0.0050 | 0.0098 |
| rs573225 | 2 | 169757541 | G | A | 0.788 | -0.012 | 0.0049 | 0.0136 |
| rs560887* | 2 | 169763148 | T | C | 0.812 | -0.010 | 0.0050 | 0.0412 |
| rs504979 | 2 | 169771859 | T | C | 0.662 | -0.010 | 0.0042 | 0.0212 |
| rs563694 | 2 | 169774071 | C | A | 0.775 | -0.009 | 0.0047 | 0.0475 |
| rs537183 | 2 | 169774646 | C | T | 0.775 | -0.009 | 0.0047 | 0.0467 |
| rs557462* | 2 | 169777595 | C | T | 0.780 | -0.010 | 0.0047 | 0.0452 |

Table S3A (Continued)

| **SNP** | **Chr** | **Position¥** | **Allele** | | **Alt Allele Frequency** | **Beta¶** | **SE** | ***p*** |
| --- | --- | --- | --- | --- | --- | --- | --- | --- |
|  |  |  | **Ref** | **Alt** |  |  |  |  |
| rs2947987 | 2 | 169778461 | T | C | 0.459 | 0.008 | 0.0039 | 0.0415 |
| rs478333 | 2 | 169779156 | A | G | 0.457 | 0.008 | 0.0039 | 0.0478 |
| rs496550 | 2 | 169779712 | C | T | 0.454 | 0.008 | 0.0039 | 0.0375 |
| rs495714 | 2 | 169779764 | T | C | 0.454 | 0.008 | 0.0039 | 0.0376 |
| rs579275 | 2 | 169780366 | C | T | 0.457 | 0.008 | 0.0039 | 0.0340 |
| rs519887 | 2 | 169780885 | C | T | 0.457 | 0.008 | 0.0039 | 0.0342 |
| rs486981 | 2 | 169782149 | A | G | 0.753 | -0.009 | 0.0046 | 0.0418 |
| rs485094 | 2 | 169782353 | A | C | 0.754 | -0.009 | 0.0046 | 0.0462 |
| rs484066 | 2 | 169782481 | A | T | 0.669 | -0.011 | 0.0042 | 0.0123 |
| rs569829* | 2 | 169782869 | T | C | 0.781 | -0.012 | 0.0047 | 0.0117 |
| rs569805 | 2 | 169782880 | A | T | 0.753 | -0.009 | 0.0045 | 0.0411 |
| rs579060 | 2 | 169783039 | G | T | 0.752 | -0.009 | 0.0045 | 0.0416 |
| rs566879 | 2 | 169783209 | A | G | 0.753 | -0.009 | 0.0045 | 0.0415 |
| rs7561903 | 2 | 169783565 | G | T | 0.849 | 0.014 | 0.0056 | 0.0130 |
| rs17540154 | 2 | 169784493 | G | A | 0.846 | 0.013 | 0.0055 | 0.0161 |
| rs508506* | 2 | 169784955 | A | C | 0.780 | -0.011 | 0.0047 | 0.0169 |
| rs503931 | 2 | 169785449 | C | A | 0.455 | 0.008 | 0.0039 | 0.0451 |
| rs34193789 | 2 | 169786287 | A | G | 0.851 | 0.013 | 0.0056 | 0.0249 |
| rs551754 | 2 | 169787686 | T | C | 0.455 | 0.008 | 0.0039 | 0.0497 |
| rs527150 | 2 | 169788039 | G | A | 0.456 | 0.008 | 0.0039 | 0.0457 |
| rs494874 | 2 | 169789306 | T | C | 0.774 | -0.012 | 0.0047 | 0.0112 |
| rs552976 | 2 | 169791438 | A | G | 0.752 | -0.012 | 0.0045 | 0.0063 |
| rs508743 | 2 | 169795287 | C | G | 0.581 | -0.010 | 0.0041 | 0.0129 |
| rs2685803 | 2 | 169796285 | G | A | 0.457 | 0.008 | 0.0040 | 0.0379 |
| rs2544367 | 2 | 169796288 | C | T | 0.457 | 0.008 | 0.0040 | 0.0370 |
| rs2685804 | 2 | 169796772 | G | A | 0.457 | 0.008 | 0.0040 | 0.0374 |
| rs2685805 | 2 | 169797060 | G | A | 0.456 | 0.009 | 0.0040 | 0.0271 |
| rs2685806 | 2 | 169797304 | G | A | 0.456 | 0.009 | 0.0040 | 0.0268 |
| rs2685807 | 2 | 169797526 | C | T | 0.558 | -0.009 | 0.0040 | 0.0256 |
| rs2685808 | 2 | 169797640 | T | G | 0.458 | 0.008 | 0.0040 | 0.0389 |
| rs1581397 | 2 | 169797652 | C | T | 0.458 | 0.008 | 0.0040 | 0.0356 |
| rs2685810 | 2 | 169797732 | C | T | 0.458 | 0.008 | 0.0040 | 0.0355 |
| rs2685811 | 2 | 169797927 | G | A | 0.457 | 0.008 | 0.0040 | 0.0349 |
| rs2685812 | 2 | 169798171 | G | A | 0.457 | 0.008 | 0.0040 | 0.0350 |
| rs2685813 | 2 | 169798439 | T | C | 0.547 | -0.009 | 0.0040 | 0.0208 |
| rs2685814 | 2 | 169798619 | T | C | 0.547 | -0.009 | 0.0040 | 0.0185 |
| rs6709087 | 2 | 169799010 | G | A | 0.845 | 0.014 | 0.0056 | 0.0125 |
| rs2250677 | 2 | 169799288 | C | T | 0.569 | -0.010 | 0.0040 | 0.0140 |
| rs853790 | 2 | 169800441 | T | A | 0.442 | 0.008 | 0.0041 | 0.0430 |

Table S3A (Continued)

| **SNP** | **Chr** | **Position¥** | **Allele** | | **Alt Allele Frequency** | **Beta¶** | **SE** | ***p*** |
| --- | --- | --- | --- | --- | --- | --- | --- | --- |
|  |  |  | **Ref** | **Alt** |  |  |  |  |
| rs853789* | 2 | 169801488 | A | G | 0.779 | -0.011 | 0.0047 | 0.0159 |
| rs860510 | 2 | 169801628 | C | A | 0.432 | 0.010 | 0.0041 | 0.0129 |
| rs853788 | 2 | 169801905 | T | C | 0.580 | -0.010 | 0.0041 | 0.0172 |
| rs853787 | 2 | 169802252 | G | T | 0.770 | -0.012 | 0.0047 | 0.0108 |
| rs853786 | 2 | 169802310 | G | A | 0.571 | -0.008 | 0.0041 | 0.0448 |
| rs862662 | 2 | 169802329 | C | A | 0.601 | -0.009 | 0.0041 | 0.0284 |
| rs853785 | 2 | 169802594 | C | T | 0.577 | -0.009 | 0.0041 | 0.0260 |
| rs853784 | 2 | 169803674 | T | C | 0.457 | 0.009 | 0.0040 | 0.0224 |
| rs853783 | 2 | 169805511 | A | C | 0.555 | -0.010 | 0.0040 | 0.0181 |
| rs7558863 | 2 | 169807362 | C | T | 0.844 | 0.012 | 0.0056 | 0.0279 |
| rs853780 | 2 | 169807482 | C | G | 0.551 | -0.008 | 0.0040 | 0.0456 |
| rs1101534 | 2 | 169807713 | T | C | 0.454 | 0.008 | 0.0040 | 0.0470 |
| rs1101533 | 2 | 169808522 | A | T | 0.552 | -0.009 | 0.0041 | 0.0287 |
| rs853779 | 2 | 169809672 | G | T | 0.548 | -0.008 | 0.0040 | 0.0439 |
| rs853777* | 2 | 169812217 | T | C | 0.780 | -0.012 | 0.0049 | 0.0141 |
| rs2943636 | 2 | 227087284 | A | G | 0.447 | 0.008 | 0.0039 | 0.0447 |
| rs2972149 | 2 | 227092150 | A | T | 0.423 | 0.008 | 0.0039 | 0.0402 |
| rs4869272* | 5 | 95539448 | C | T | 0.750 | -0.010 | 0.0045 | 0.0308 |
| rs13179048* | 5 | 95542726 | A | C | 0.759 | -0.009 | 0.0045 | 0.0376 |
| rs12186664* | 5 | 95630225 | T | A | 0.751 | -0.009 | 0.0044 | 0.0480 |
| rs13162665* | 5 | 95642519 | G | T | 0.758 | -0.009 | 0.0045 | 0.0494 |
| rs11765149 | 7 | 14877519 | C | G | 0.885 | -0.012 | 0.0062 | 0.0484 |
| rs994369 | 7 | 14898152 | C | T | 0.475 | -0.008 | 0.0040 | 0.0423 |
| rs217554 | 7 | 14905933 | A | G | 0.490 | 0.009 | 0.0040 | 0.0311 |
| rs10276674 | 7 | 14922007 | C | T | 0.649 | -0.011 | 0.0045 | 0.0117 |
| rs7800343 | 7 | 15021223 | A | G | 0.727 | 0.011 | 0.0044 | 0.0138 |
| rs12670968 | 7 | 15026787 | A | G | 0.751 | 0.010 | 0.0045 | 0.0224 |
| rs6461145 | 7 | 15026872 | G | A | 0.731 | 0.010 | 0.0043 | 0.0184 |
| rs17168579 | 7 | 15038077 | G | A | 0.711 | 0.009 | 0.0044 | 0.0498 |
| rs12113083 | 7 | 15051943 | C | G | 0.597 | 0.008 | 0.0040 | 0.0497 |
| rs11514706 | 7 | 15059272 | C | A | 0.605 | 0.008 | 0.0041 | 0.0446 |
| rs10231021 | 7 | 15060429 | A | T | 0.557 | 0.008 | 0.0040 | 0.0427 |
| rs6947830 | 7 | 15064984 | G | A | 0.478 | -0.008 | 0.0039 | 0.0474 |
| rs4719433 | 7 | 15065003 | T | C | 0.478 | -0.008 | 0.0039 | 0.0474 |
| rs6967891 | 7 | 15065074 | T | C | 0.478 | -0.008 | 0.0039 | 0.0467 |
| rs76323047* | 7 | 44185955 | G | A | 0.848 | 0.014 | 0.0057 | 0.0117 |
| rs2041547 | 7 | 44200884 | T | C | 0.555 | -0.008 | 0.0040 | 0.0491 |
| rs2080033 | 7 | 44204322 | G | A | 0.554 | -0.009 | 0.0038 | 0.0219 |
| rs7793213 | 7 | 44204994 | G | A | 0.523 | -0.008 | 0.0038 | 0.0342 |

Table S3A (Continued)

| **SNP** | **Chr** | **Position¥** | **Allele** | | **Alt Allele Frequency** | **Beta¶** | **SE** | ***p*** |
| --- | --- | --- | --- | --- | --- | --- | --- | --- |
|  |  |  | **Ref** | **Alt** |  |  |  |  |
| rs2908291 | 7 | 44213222 | A | T | 0.537 | -0.009 | 0.0039 | 0.0230 |
| rs2284776 | 7 | 44216754 | C | T | 0.510 | -0.008 | 0.0039 | 0.0291 |
| rs2244164 | 7 | 44217126 | C | T | 0.522 | -0.009 | 0.0038 | 0.0269 |
| rs1303722 | 7 | 44219074 | T | C | 0.521 | -0.008 | 0.0038 | 0.0291 |
| rs1990458 | 7 | 44222802 | T | C | 0.596 | -0.013 | 0.0045 | 0.0044 |
| rs730497* | 7 | 44223721 | A | G | 0.799 | 0.014 | 0.0048 | 0.0036 |
| rs2908289* | 7 | 44223942 | A | G | 0.794 | 0.014 | 0.0047 | 0.0044 |
| rs3808319 | 7 | 44224851 | A | G | 0.632 | -0.010 | 0.0048 | 0.0289 |
| rs2268570 | 7 | 44226090 | G | C | 0.586 | -0.012 | 0.0043 | 0.0056 |
| rs2971670 | 7 | 44226101 | T | C | 0.800 | 0.014 | 0.0048 | 0.0036 |
| rs1799884 | 7 | 44229068 | T | C | 0.800 | 0.014 | 0.0048 | 0.0035 |
| rs2971669 | 7 | 44231778 | T | C | 0.727 | 0.012 | 0.0048 | 0.0159 |
| rs6975024* | 7 | 44231886 | C | T | 0.810 | 0.014 | 0.0050 | 0.0046 |
| rs741037* | 7 | 44232833 | A | G | 0.790 | 0.014 | 0.0048 | 0.0033 |
| rs2908286* | 7 | 44234737 | T | C | 0.798 | 0.014 | 0.0048 | 0.0028 |
| rs4607517* | 7 | 44235668 | A | G | 0.806 | 0.014 | 0.0050 | 0.0041 |
| rs12056308* | 7 | 44239034 | A | G | 0.789 | 0.015 | 0.0048 | 0.0027 |
| rs1985469* | 7 | 44240324 | T | A | 0.733 | 0.013 | 0.0044 | 0.0048 |
| rs1004558* | 7 | 44240407 | T | C | 0.729 | 0.012 | 0.0044 | 0.0059 |
| rs2971668* | 7 | 44243438 | C | G | 0.727 | 0.012 | 0.0044 | 0.0045 |
| rs2971667* | 7 | 44245060 | C | T | 0.723 | 0.012 | 0.0044 | 0.0051 |
| rs917793* | 7 | 44245853 | T | A | 0.724 | 0.012 | 0.0044 | 0.0043 |
| rs2908282* | 7 | 44248828 | A | G | 0.728 | 0.012 | 0.0043 | 0.0042 |
| rs983309 | 8 | 9177732 | T | G | 0.808 | 0.010 | 0.0049 | 0.0411 |
| rs6984305 | 8 | 9178268 | A | T | 0.817 | 0.011 | 0.0050 | 0.0255 |
| rs4300038 | 8 | 118217915 | A | G | 0.739 | -0.010 | 0.0046 | 0.0400 |
| rs11774700 | 8 | 118220270 | C | T | 0.735 | -0.011 | 0.0046 | 0.0215 |
| rs4258313 | 10 | 113032398 | T | G | 0.893 | -0.012 | 0.0062 | 0.0432 |
| rs7071574 | 10 | 113033128 | A | G | 0.893 | -0.013 | 0.0062 | 0.0414 |
| rs12784552 | 10 | 113036354 | G | A | 0.893 | -0.013 | 0.0062 | 0.0391 |
| rs12792753* | 11 | 92668975 | C | T | 0.692 | 0.009 | 0.0041 | 0.0328 |
| rs1387153 | 11 | 92673828 | T | C | 0.753 | 0.012 | 0.0046 | 0.0087 |
| rs2121651 | 11 | 92677698 | G | T | 0.707 | 0.009 | 0.0042 | 0.0447 |
| rs6483206 | 11 | 92678397 | T | C | 0.709 | 0.009 | 0.0043 | 0.0439 |
| rs2121648 | 11 | 92679433 | T | G | 0.752 | 0.012 | 0.0046 | 0.0107 |
| rs2121647 | 11 | 92679439 | C | G | 0.706 | 0.009 | 0.0042 | 0.0396 |
| rs11523890 | 11 | 92679778 | T | C | 0.706 | 0.009 | 0.0042 | 0.0398 |
| rs10830956 | 11 | 92681013 | T | C | 0.750 | 0.012 | 0.0046 | 0.0103 |
| rs10765572 | 11 | 92681234 | C | T | 0.750 | 0.012 | 0.0046 | 0.0101 |

Table S3A (Continued)

| **SNP** | **Chr** | **Position¥** | **Allele** | | **Alt Allele Frequency** | **Beta¶** | **SE** | ***p*** |
| --- | --- | --- | --- | --- | --- | --- | --- | --- |
|  |  |  | **Ref** | **Alt** |  |  |  |  |
| rs11020114 | 11 | 92682604 | C | T | 0.707 | 0.009 | 0.0043 | 0.0333 |
| rs10830957 | 11 | 92683073 | T | C | 0.709 | 0.009 | 0.0043 | 0.0289 |
| rs10765573 | 11 | 92683332 | A | T | 0.707 | 0.009 | 0.0042 | 0.0364 |
| rs10830958 | 11 | 92683955 | G | T | 0.707 | 0.009 | 0.0043 | 0.0357 |
| rs10830959 | 11 | 92685116 | G | A | 0.750 | 0.012 | 0.0046 | 0.0088 |
| rs10830960 | 11 | 92688012 | T | A | 0.707 | 0.009 | 0.0043 | 0.0304 |
| rs10741452 | 11 | 92689350 | G | A | 0.706 | 0.009 | 0.0043 | 0.0293 |
| rs7936247 | 11 | 92690032 | T | G | 0.707 | 0.009 | 0.0043 | 0.0289 |
| rs11020124 | 11 | 92690661 | C | T | 0.753 | 0.013 | 0.0047 | 0.0059 |
| rs4331050 | 11 | 92696014 | G | T | 0.368 | -0.008 | 0.0040 | 0.0461 |
| rs10466351* | 11 | 92697981 | T | C | 0.637 | 0.009 | 0.0041 | 0.0236 |
| rs10830962 | 11 | 92698427 | C | G | 0.368 | -0.008 | 0.0040 | 0.0459 |
| rs7941837* | 11 | 92699666 | A | T | 0.379 | -0.008 | 0.0040 | 0.0375 |
| rs7945617* | 11 | 92700287 | T | C | 0.380 | -0.008 | 0.0040 | 0.0362 |
| rs10830963* | 11 | 92708710 | G | C | 0.775 | 0.013 | 0.0047 | 0.0062 |

Alt, alternative; Chr, chromosome; FG, fasting glucose; GWA, genome-wide association; Ref, reference; SE, standard error; SHARe, SNP Health Association Resource; SNP, single-nucleotide polymorphism; WHI, Women’s Health Initiative.

¥ GRCh 37 coordinated.

¶ Beta from regression analysis was adjusted for 10 genetic principal components as well as age.

* SNPs shared by African American women are indicated.

1. Among the GWA FI-SNPs derived from previous GWA studies, 1 SNP nominally was associated with the fasting level of naturally log-transformed insulin in the WHI SHARe data.

| **SNP** | **Chr** | **Position¥** | **Allele** | | **Alt Allele Frequency** | **Beta¶** | **SE** | ***p*** |
| --- | --- | --- | --- | --- | --- | --- | --- | --- |
|  |  |  | **Ref** | **Alt** |  |  |  |  |
| rs4846567 | 1 | 219750717 | T | G | 0.572 | 0.038 | 0.0195 | 0.0489 |

Alt, alternative; Chr, chromosome; FI, fasting insulin; GWA, genome-wide association; Ref, reference; SE, standard error; SHARe, SNP Health Association Resource; SNP, single-nucleotide polymorphism; WHI, Women’s Health Initiative.

¥ GRCh 37 coordinated.

¶ Beta from regression analysis was adjusted for 10 genetic principal components as well as age.

1. Cox regressions of 27 genome-wide SNPs associated with FG, predicting CRC risk at significance nominally and after multiple comparison correction.

| **SNP** | **Chr** | **Position¥** | **Allele** | | **HR¶ (95% CI)** | **SE** | ***p*** |
| --- | --- | --- | --- | --- | --- | --- | --- |
|  |  |  | **Ref** | **Alt** |  |  |  |
| rs4665378 | 2 | 27548038 | C | A | 6.509 (2.422 - 17.497) | 0.5045 | 0.0002**†** |
| rs1049817 | 2 | 27550967 | G | A | 6.485 (2.417 - 17.396) | 0.5035 | 0.0002**†** |
| rs10205219 | 2 | 27568565 | C | T | 6.570 (2.451 - 17.614) | 0.5031 | 0.0002**†** |
| rs4665969 | 2 | 27574953 | C | T | 6.972 (2.613 - 18.604) | 0.5008 | 0.0001**†** |
| rs6760828 | 2 | 27579231 | C | T | 6.800 (2.497 - 18.522) | 0.5112 | 0.0002**†** |
| rs1528533 | 2 | 27595756 | C | G | 8.067 (2.770 - 23.488) | 0.5453 | 0.0001**†** |
| rs4665972 | 2 | 27598097 | T | C | 0.161 (0.045 - 0.573) | 0.6473 | 0.0048 |
| rs1060525 | 2 | 27635582 | G | A | 7.972 (2.732 - 23.263) | 0.5464 | 0.0001**†** |
| rs4665976 | 2 | 27640325 | G | A | 7.944 (2.732 - 23.099) | 0.5446 | 0.0001**†** |
| rs1728922 | 2 | 27644464 | C | A | 7.848 (2.704 - 22.779) | 0.5437 | 0.0002**†** |
| rs780100 | 2 | 27652153 | T | G | 7.821 (2.714 - 22.538) | 0.5400 | 0.0001**†** |
| rs704791 | 2 | 27657167 | C | T | 7.718 (2.691 - 22.138) | 0.5376 | 0.0001**†** |
| rs780102 | 2 | 27659491 | C | T | 7.705 (2.687 - 22.092) | 0.5374 | 0.0001**†** |
| rs780104 | 2 | 27677691 | A | G | 7.288 (2.595 - 20.469) | 0.5269 | 0.0002**†** |
| rs704795 | 2 | 27716494 | A | G | 6.427 (2.403 - 17.187) | 0.5019 | 0.0002**†** |
| rs1260320 | 2 | 27722416 | A | G | 6.402 (2.401 - 17.069) | 0.5003 | 0.0002**†** |
| rs1260326 | 2 | 27730940 | T | C | 0.332 (0.127 - 0.870) | 0.4917 | 0.0249 |
| rs6547692 | 2 | 27734972 | G | A | 0.242 (0.091 - 0.645) | 0.5002 | 0.0046 |
| rs780096 | 2 | 27741072 | C | G | 0.245 (0.094 - 0.641) | 0.4902 | 0.0041 |
| rs780095 | 2 | 27741105 | A | G | 0.250 (0.095 - 0.658) | 0.4935 | 0.0050 |
| rs780094 | 2 | 27741237 | T | C | 0.318 (0.122 - 0.829) | 0.4895 | 0.0191 |
| rs780093 | 2 | 27742603 | T | C | 0.336 (0.130 - 0.866) | 0.4828 | 0.0239 |
| rs11681351 | 2 | 27743423 | A | G | 5.396 (2.184 - 13.333) | 0.4615 | 0.0003**†** |
| rs1260333 | 2 | 27748624 | A | G | 0.268 (0.105 - 0.688) | 0.4801 | 0.0062 |
| rs1313566 | 2 | 27748904 | G | A | 0.270 (0.106 - 0.688) | 0.4778 | 0.0061 |
| rs11127048 | 2 | 27752463 | G | A | 0.308 (0.116 - 0.819) | 0.4987 | 0.0183 |
| rs6753534 | 2 | 27752871 | C | T | 0.269 (0.102 - 0.707) | 0.4943 | 0.0078 |

Alt, alternative; Chr, chromosome; CI, confidence interval; CRC, colorectal cancer; FG, fasting glucose; HR, hazard ratio; Ref, reference; SE, standard error; SNP, single-nucleotide polymorphism.

¥ GRCh 37 coordinated.

¶ HR from regression analysis was adjusted for 10 genetic principal components as well as age.

† *p* value indicates that the corresponding SNP is statistically significant after the Bonferroni correction for multiple comparisons.
