# Supplementary material for: Synergistic Effects of Genetic Variants of Glucose Homeostasis and Lifelong Exposures to Cigarette Smoking, Female Hormones, and Dietary Fat Intake on Primary Colorectal Cancer Development in African and Hispanic/Latino American Women
Source: Front Oncol. 2021 Oct 7;11:760243. doi: 10.3389/fonc.2021.760243 (PMC8529283; doi:10.3389/fonc.2021.760243)
Supplement: Supplementary file 1 [file DataSheet_1.zip › Table S4.RSF.docx]

Table S4. RSF analysis: predictive values of genetic and lifestyle factors for colorectal cancer risk

1. African American women: top 10 genetic variables

| **SNP*** | **Minimal Depth†** | **VIMP** | **Incremental Error**¶ | **Drop Error§** |
| --- | --- | --- | --- | --- |
| *PCSK1* rs9285019¥ | 2.0768 | 0.0208 | 0.5285 | -0.0285 |
| *PCSK1* rs6234 | 2.1718 | -0.0106 | 0.4620 | 0.0666 |
| *PCSK1* rs193069188 | 2.3778 | -0.0027 | 0.4482 | 0.0137 |
| *PCSK1* rs13169290 | 2.5490 | -0.0078 | 0.4854 | -0.0372 |
| *GCK* rs2908286 | 2.5576 | 0.0022 | 0.4565 | 0.0289 |
| *PCSK1* rs17085665 | 2.7276 | -0.0201 | 0.4651 | -0.0086 |
| GCK rs2971670 | 3.0496 | 0.0018 | 0.4584 | 0.0067 |
| *GCK* rs730497 | 3.3814 | 0.0003 | 0.4623 | -0.0038 |
| *YKT6* rs2908282 | 4.3522 | -0.0058 | 0.4637 | -0.0014 |
| *GCK* rs1799884 | 7.3040 | -0.0026 | 0.4641 | -0.0004 |

SNP, single-nucleotide polymorphism; RSF, random survival forest; VIMP, variable of importance.

* SNPs ordered by minimal depth.

† Minimal depth is the predictive value of a variable estimated from the nested RSF models, with a lower value being likely to have a greater impact on prediction.

¶ Incremental error was calculated using the nested sequence of models starting with the top variable, followed by the model with the top 2 variables, then the model with the top 3 variables, and so on. For example, the third error was computed from the third nested model, including the first, second, and third variables.

**§** Drop error of the variable was calculated by the difference between the errors of a prior and the corresponding variable from the nested models. For example, the drop error of the second variable was estimated by the difference between the errors from the first and second nested models. The error for the null model is set at 0.5; thus, the drop error for the first variable was obtained by subtracting the error (0.5285) from 0.5.

¥ SNP was selected as the most predictive genetic markers on the basis of multimodal predictive values.

1. African American women: top 14 lifestyle variables

| **Lifestyle variable*** | **Minimal Depth†** | **VIMP** | **Incremental Error¶** | **Drop Error§** |
| --- | --- | --- | --- | --- |
| Years as a regular smoker¥ | 3.8566 | 0.0182 | 0.4977 | 0.0023 |
| Percent calories from PFA/day¥ | 4.0366 | 0.0067 | 0.4644 | 0.0333 |
| Age at menopause¥ | 4.1294 | 0.0048 | 0.4514 | 0.0130 |
| Age at enrollment¥ | 4.2214 | 0.0027 | 0.3986 | 0.0528 |
| Duration of oral contraceptive use¥ | 4.3218 | 0.0015 | 0.3777 | 0.0209 |
| Dietary calcium | 4.4198 | -0.0002 | 0.3764 | 0.0013 |
| Dietary total sugars¥ | 4.6352 | 0.0015 | 0.3699 | 0.0065 |
| Number of pregnancies | 4.6914 | < 0.0001 | 0.3676 | 0.0023 |
| Body mass index | 4.9974 | -0.0021 | 0.3893 | -0.0217 |
| Percent calories from protein/day | 5.1830 | 0.0002 | 0.3910 | -0.0017 |
| Waist-to-hip ratio | 5.2538 | 0.0003 | 0.3912 | -0.0002 |
| Age at menarche | 5.2840 | -0.0024 | 0.3972 | -0.0061 |
| Percent calories from SFA/day | 5.3990 | 0.0003 | 0.3988 | -0.0016 |
| Waist circumference | 5.5214 | -0.0038 | 0.4100 | -0.0112 |

PFA, polyunsaturated fatty acid; SFA, saturated fatty acid; VIMP, variable of importance.

* Variables ordered by minimal depth.

† Minimal depth is the predictive value of a variable estimated from the nested RSF models, with a lower value being likely to have a greater impact on prediction.

¶ Incremental error was calculated using the nested sequence of models starting with the top variable, followed by the model with the top 2 variables, then the model with the top 3 variables, and so on. For example, the third error was computed from the third nested model, including the first, second, and third variables.

**§** Drop error of the variable was calculated by the difference between the errors of a prior and the corresponding variable from the nested models. For example, the drop error of the second variable was estimated by the difference between the errors from the first and second nested models. The error for the null model is set at 0.5; thus, the drop error for the first variable was obtained by subtracting the error (0.4977) from 0.5.

¥ Variables were selected as the most predictive lifestyle factors on the basis of multimodal predictive values.

1. Hispanic American women: top 12 genetic variables

| **SNP*** | **Minimal Depth†** | **VIMP** | **Incremental Error**¶ | **Drop Error§** |
| --- | --- | --- | --- | --- |
| *IFT172* rs780104¥ | 4.3684 | 0.0265 | 0.2936 | 0.2064 |
| *GCKR* rs6753534¥ | 4.4200 | 0.0170 | 0.1825 | 0.1111 |
| *NRBP1* rs704791¥** | 4.4804 | 0.0175 | 0.1952 | -0.0126 |
| *NRBP1* rs780102¥** | 4.7954 | 0.0142 | 0.2352 | -0.0401 |
| *GCKR* rs11127048 | 4.8850 | -0.0040 | 0.2270 | 0.0082 |
| *NRBP1* rs780100¥** | 4.9050 | 0.0176 | 0.2522 | -0.0252 |
| *GCKR* rs1260326 | 4.9306 | -0.0036 | 0.2404 | 0.0118 |
| *NRBP1* rs1728922 | 4.9784 | 0.0121 | 0.2375 | 0.0029 |
| *GCKR* rs1260333 | 5.0484 | 0.0125 | 0.2310 | 0.0065 |
| *SNX17* rs4665972 | 5.1072 | -0.0185 | 0.2514 | -0.0204 |
| *PPM1G* rs4665976 | 5.3736 | -0.0054 | 0.2525 | -0.0011 |
| *MPV17* rs4665378 | 5.3750 | 0.0080 | 0.2597 | -0.0072 |

SNP, single-nucleotide polymorphism; VIMP, variable of importance.

* SNPs ordered by minimal depth.

† Minimal depth is the predictive value of a variable estimated from the nested RSF models, with a lower value being likely to have a greater impact on prediction.

¶ Incremental error was calculated using the nested sequence of models starting with the top variable, followed by the model with the top 2 variables, then the model with the top 3 variables, and so on. For example, the third error was computed from the third nested model, including the first, second, and third variables.

**§** Drop error of the variable was calculated by the difference between the errors of a prior and the corresponding variable from the nested models. For example, the drop error of the second variable was estimated by the difference between the errors from the first and second nested models. The error for the null model is set at 0.5; thus, the drop error for the first variable was obtained by subtracting the error (0.2936) from 0.5.

¥ SNPs were selected as the most predictive genetic markers on the basis of multimodal predictive values.

** Due to the high linkage disequilibrium within the 3 SNPs in *NRBP1*, the SNP (rs704791) with the strongest predictability (i.e., lowest minimal depth, highest VIMP, and smallest error) was selected as an index and carried forward to the analysis for the association with colorectal cancer risk.

1. Hispanic American women: top 18 lifestyle variables

| **Lifestyle variable*** | **Minimal Depth†** | **VIMP** | **Incremental Error¶** | **Drop Error§** |
| --- | --- | --- | --- | --- |
| Percent calories from MFA/day¥ | 4.1646 | 0.0211 | 0.4021 | 0.0979 |
| Number of cigarettes/day¥ | 4.6240 | 0.0073 | 0.4755 | -0.0733 |
| Age at menopause¥ | 4.9540 | 0.0136 | 0.4346 | 0.0409 |
| Percent calories from SFA/day¥ | 5.2530 | 0.0018 | 0.4164 | 0.0181 |
| Percent calories from PFA/day¥ | 5.3560 | 0.0033 | 0.4104 | 0.0060 |
| Dietary vitamin K¥ | 5.3944 | 0.0023 | 0.4279 | -0.0175 |
| One or both ovaries removed | 5.4612 | -0.0018 | 0.4123 | 0.0156 |
| Dietary calcium | 5.5114 | -0.0011 | 0.4123 | 0.0000 |
| Age at enrollment | 5.5578 | -0.0011 | 0.3931 | 0.0192 |
| Duration of oral contraceptive use | 5.5746 | 0.0035 | 0.3887 | 0.0043 |
| Dietary alcohol | 5.6000 | 0.0013 | 0.3925 | -0.0038 |
| Daily fruit consumption | 5.6330 | 0.0010 | 0.3816 | 0.0109 |
| Cardiovascular disease ever | 5.6744 | 0.0067 | 0.3470 | 0.0347 |
| Percent calories from protein | 5.7358 | 0.0004 | 0.3684 | -0.0214 |
| Dietary total sugars | 5.8268 | -0.0032 | 0.3809 | -0.0125 |
| Dietary fiber | 5.8418 | -0.0001 | 0.4002 | -0.0194 |
| Categorization of alcohol intake | 5.9358 | 0.0015 | 0.4030 | -0.0027 |
| Body mass index | 5.9546 | -0.0011 | 0.4181 | -0.0151 |

MFA, monounsaturated fatty acid; PFA, polyunsaturated fatty acid; SFA, saturated fatty acid; VIMP, variable of importance.

* Variables ordered by minimal depth.

† Minimal depth is the predictive value of a variable estimated from the nested RSF models, with a lower value being likely to have a greater impact on prediction.

¶ Incremental error was calculated using the nested sequence of models starting with the top variable, followed by the model with the top 2 variables, then the model with the top 3 variables, and so on. For example, the third error was computed from the third nested model, including the first, second, and third variables.

**§** Drop error of the variable was calculated by the difference between the errors of a prior and the corresponding variable from the nested models. For example, the drop error of the second variable was estimated by the difference between the errors from the first and second nested models. The error for the null model is set at 0.5; thus, the drop error for the first variable was obtained by subtracting the error (0.4021) from 0.5.

¥ Variables were selected as the most predictive lifestyle factors on the basis of multimodal predictive values.
