# Supplementary material for: Synergistic Effects of Genetic Variants of Glucose Homeostasis and Lifelong Exposures to Cigarette Smoking, Female Hormones, and Dietary Fat Intake on Primary Colorectal Cancer Development in African and Hispanic/Latino American Women
Source: Front Oncol. 2021 Oct 7;11:760243. doi: 10.3389/fonc.2021.760243 (PMC8529283; doi:10.3389/fonc.2021.760243)
Supplement: Supplementary file 1 [file DataSheet_1.zip › Table S5.individual HRs.docx]

Table S5. Multiple Cox regression results: individual SNPs and lifestyle factors, adjusted for each other, predicting colorectal cancer risk

1. African American women

| **SNP/lifestyle factor†** | **Allele** | **/** | **cutoff value** | **HR (95% CI)** | ***p*** |
| --- | --- | --- | --- | --- | --- |
|  | **Ref** | **vs.** | **Alt/effect value** |  |  |
| *PCSK1* rs9285019 | TT | vs. | TC+CC | **1.59 (1.00 – 2.52)** | **0.0490** |
| Years as a regular smoker | < 20 years | vs. | ≥ 20 years€ | **2.84 (1.66 – 4.84)** | **0.0001*** |
| Percent calories from PFA/day | > 6.8% | vs. | ≤ 6.8% | 1.16 (0.72 – 1.85) | 0.5393 |
| Age at menopause | ≤ 42 years | vs. | > 42 years | 1.35 (0.77 – 2.35) | 0.2910 |
| Age at enrollment | 50 – 55 years | vs. | 56 – 79 years | 1.70 (0.89 – 3.25) | 0.1061 |
| Duration of oral contraceptive use | 0 – 4 years | vs. | 5 – 37 years | 1.39 (0.73 – 2.65) | 0.3118 |
| Dietary total sugars | ≤ 60.5g | vs. | > 60.5g | 1.26 (0.68 – 2.35) | 0.4683 |

Alt, alternative allele; CI, confidence interval; HR, hazard ratio; PFA, polyunsaturated fatty acid; Ref, reference allele or reference value; SNP, single-nucleotide polymorphism. Numbers in bold face are statistically significant.

† Modeled SNP and lifestyle factors were obtained from random survival forest analysis with a multimodal approach.

€ Years as a regular smoker was measured by a 10-year interval ranging from < 5 years to ≥ 50 years.

* *p* value was corrected for multiple comparisons via the Benjamini-Hochberg approach.

1. Hispanic American women

| **SNP/lifestyle factor†** | **Allele** | **/** | **cutoff value** | **HR (95% CI)** **€** | ***p*** |
| --- | --- | --- | --- | --- | --- |
|  | **Ref** | **vs.** | **Alt/effect value** |  |  |
| *IFT172* rs780104 | AA + AG | vs. | GG | **9.57 (3.08 – 29.67)** | **9.20E-05** |
| *GCKR* rs6753534 | CT + TT | vs. | CC | **3.38 (1.23 – 9.31)** | **1.83E-02** |
| *NRBP1* rs704791 | CC + CT | vs. | TT | **9.59 (3.09 – 29.75)** | **9.03E-05** |
| Percent calories from MFA/day | ≤ 15.9% | vs. | > 15.9% | **6.34 (1.83 – 21.94)** | **0.0035*** |
| Number of cigarettes/day | ≤ 24 cigarettes | vs. | ≥ 25 cigarettes | **8.62 (1.89 – 39.34)** | **0.0054*** |
| Age at menopause | > 38 years | vs. | ≤ 38 years | 2.19 (0.62 – 7.80) | 0.2256 |
| Percent calories from SFA/day | ≤ 12.4% | vs. | > 12.4% | 0.91 (0.28 – 3.00) | 0.8815 |
| Percent calories from PFA/day | > 4.7% | vs. | ≤ 4.7% | 2.61 (0.71 – 9.59) | 0.1486 |
| Dietary vitamin K | > 55.6mg | vs. | ≤ 55.6mg | **2.97 (1.03 – 8.56)** | **0.0439** |

Alt, alternative allele; CI, confidence interval; HR, hazard ratio; MFA, monounsaturated fatty acid; PFA, polyunsaturated fatty acid; Ref, reference allele or reference value; SFA, saturated fatty acid; SNP, single-nucleotide polymorphism. Numbers in bold face are statistically significant.

† Modeled SNPs and lifestyle factors were obtained from random survival forest analysis with a multimodal approach.

€ Due to the missing value in the multivariate analysis within the SNPs, the HRs of the SNPs were obtained from the univariate analysis. The HRs of the lifestyle factors were adjusted for each other as well as for the combined number of alternative alleles (null/1 vs. 2+) from the SNPs.

* *p* values were corrected for multiple comparisons via the Benjamini-Hochberg approach.
